# Microsatellites reveal divergence in population genetic diversity, and structure of *osyris lanceolata* (santalaceae) in Uganda and Kenya

**DOI:** 10.1186/s12862-023-02182-2

**Published:** 2023-12-08

**Authors:** Ben Belden Mugula, S. F. Omondi, Manuel Curto, Samuel Kuria Kiboi, James Ireri Kanya, Anthony Egeru, Paul Okullo, Harald Meimberg

**Affiliations:** 1 Department of Biology, Faculty of Science and Technology, University of Nairobi, Nairobi, Kenya; 2 Department of Agriculture and Environmental Sciences, Bugema University, Kampala, Uganda; 3 Kenya Forestry Research Institute (KEFRI), Nairobi, Kenya; 4 CIBIO—Research Center in Biodiversity and Genetic Resources, University of Porto, Vairão, 4485-661 Portugal; 5 BIOPOLIS Program in Genomics, Biodiversity and Land Planning, CIBIO, Campus de Vairão, Vairão, 4485-661 Portugal; 6 College of Environmental and Agricultural Sciences, Makerere University, P.O. Box 7062, Kampala, Uganda; 7 Nabuin Zonal Agricultural Research & Development Institute, National Agricultural Research Organisation (NARO), P.O. Box 132, Moroto, Uganda; 8 Department of Integrative Biology and Biodiversity Research, Institute of Integrative Nature Conservation Research, University of Natural Resources and Life Sciences, Vienna, Austria

**Keywords:** Genetic diversity, Population genetics, Genetic differentiation, Osyris lanceolata, Population bottlenecks

## Abstract

**Background:**

*Osyris lanceolata* (Hochst. & Steud.) (Santalaceae) is a multipurpose plant highly valued culturally and economically in Africa. However, *O. lanceolata* populations have rapidly dwindled in East Africa due to overexploitation and this is believed to cause further consequences on the species’ genetic diversity and structure within the region. Information regarding a species’ genetic diversity and structure is necessary for conservation but this is currently lacking for *O. lanceolata* in Uganda and Kenya. Lack of adequate scientific data hinders conservation efforts hence threatening the species survival and livelihoods. This study investigated patterns in genetic diversity and structure of *O. lanceolata* in Uganda and Kenya. Ten polymorphic microsatellite loci were used to genotype 210 individuals: 96 from Ugandan and 114 from Kenyan populations.

**Results:**

All populations were highly polymorphic (80–100% polymorphism). A genetic differentiation was found between Kenyan and Ugandan populations. The highest genetic differentiation was among individuals and the least among populations. The Kenyan populations showed higher genetic diversity than Ugandan populations. The Ugandan populations showed more marker deviations from Hardy-Weinberg equilibrium and inbreeding coefficient. Two populations showed evidence of going through a recent bottleneck. There was significant genetic differentiation and structuring at higher K values into larger clusters and observed admixture between populations. The populations were significantly isolated by altitude as opposed to distance and climatic variables. Main barriers were associated with altitude differences. The data supports the idea of long-distance gene-flow between high altitude populations in both countries.

**Conclusion:**

The divergence in genetic structure suggests unrecognised taxonomic units within *O. lanceolata* which are characteristic to lower altitudes and higher altitudes including most Kenyan populations with divergent evolutionary patterns. Geographical barriers and environmental gradients could have influenced this genetic divergence, and such patterns may escalate the species microevolutionary processes into full allopatric speciation. Further investigations into the species’ genetic admixture and emerging taxonomic units are necessary to guide conservation strategies in the region.

**Supplementary Information:**

The online version contains supplementary material available at 10.1186/s12862-023-02182-2.

## Introduction

East African sandalwood (*Osyris lanceolata*, Hochst. & Steud., Santalaceae) is a valuable commercial species because of its aromatic wood and essential oils used in perfumery and pharmaceutical industries [1–4]. The species is widely distributed in Africa, Asia, Europe and the Socotra Islands with an unknown origin [2–5]. It is used as medicine for candidiasis, malaria, diarrhoea, chest pain and fever in Africa [2, 6–8]. The oil has chemopreventive properties for managing eruptive skin [9], inflammatory diseases, and urinary infections. The species bark and root have potential in phytoremediation [10], and they provide a red dye for skin tanning.

*Osyris lanceolata* is a dioecious plant, existing in two different sexual forms as male and female plant [11]. Being dioecious, it exhibits cross pollination, and its germination largely depends on dispersal by frugivorous birds, mammals, and wind [1, 11–13]. The species has challenges in achieving successful pollination and usually assisted pollination enhances its success by over 39% [12]. *Osyris lanceolata* has been characterised by lower rate of seedling recruitment due to seedling mortality attributed to browsing and grazing, poor seed viability due to fruit and seed destruction by pests such as *Dismiqus sp* [12]. In most cases, the species regenerates vegetatively through coppicing, and sprouting of its root tubers [12]. These biological attributes generally drive the species genetic diversity and structuring at local and regional scales.

The utilisation of *O. lanceolata* oils in the perfumery industries increased after a decline in the Indian (*Santalum alba*) and Australian (*Santalum spicutum*) sandalwood populations in the 1990s, which shifted pressure to *O. lanceolata* populations in East Africa, leading to overexploitation of the species [3, 13]. Several reports of illegal trade and destructive harvesting (whole plant uprooting) of the species have been reported in African countries with no success in conservation strategies to date despite emerging initiatives to commercially propagate the species by Kenyan farmers ( [1–3, 13–17]. However, such conservation initiatives have been hindered by a number of factors such as lack of propagation materials, poor identification of suitable provenances for propagation and the dwindling sources of the species germplasm in the wild habitats [7]. Unfortunately, such ex-situ initiatives for the species propagation on a commercial scale are still limited to very few countries (Kenya and Tanzania) in East Africa, and completely non-existent in other countries such as Uganda, Sudan and Rwanda where the species occurs [2].

*Osyris lanceolata* populations are already being affected by multiple stressors, such as habitat loss through urbanisation and deforestation and uncontrolled timber harvesting for multiple purposes, such as charcoal production and construction material [12]. The increasing demand for essential oils added extra pressure to *O. lanceolata* populations in Africa, hence threatening its survival [13]. In addition, certain aspects of *O. lanceolata* biology, such as seed germination failure, may contribute to a faster decline of this species, which has extremely lower levels of seed germination success in East Africa [12, 18–22]. Also, *O. lanceolata* is a hemiparasitic plant, and its survival, is also affected by the presence of hosts such as *Rhus natalensis*, which happens to be threatened by multiple stressors such as nonregulated wood harvesting [12, 21]. Furthermore, the species conservation efforts have not been successful to date because of limited planting materials (seedlings), poor propagation techniques, unknown provenances, seedbanks and poor understanding of species ecology and genetics. In fact, *O. lanceolata* is one of the least genetically and ecologically studied species among African trees [2]. Even with the current rapid advancement in genetic technology [2, 23], the species has limited genetic studies, particularly in East Africa and this hinders effective management strategies due to over synonymisation and an unclear recognition of the species taxonomic units [2].

The destructive harvesting of *O. lanceolata*, which is a dioecious plant, also increases the risk of extreme variation in species sex ratios, population decline, and alterations in dispersion, density, and distribution patterns. These factors can ultimately contribute to the reduction in genetic diversity with long-term consequences on species survival and resilience to changing environmental conditions [22–24]. The diversity of alleles and genotypes provides a basis for species survival, evolution and genetic adaptive ability to change, hence making populations more resilient to environmental changes [25]. Therefore, a decline in species genetic diversity would weaken the species’ evolutionary potential, resilience, and adaptation, hence predisposing the species to a higher risk of extinction [25–30]. This loss of genetic diversity needs to be quantified to understand the species genetic patterns and develop efficient conservation measures.

High-throughput sequencing technologies have facilitated the development of molecular markers such as microsatellites that are extremely informative in the assessment of population genetics and therefore evaluate the impact of human activities on evolutionary processes [31–33]. Due to their high mutation rate and mostly neutral nature, microsatellite markers are especially informative in retrieving genetic variation patterns within and among populations of the same species that result from demographic responses to anthropogenic and environmental impacts [25, 32]. The unravelling of species genetic diversity and structure explains population dynamics and trends in evolutionary processes as a basis for sound conservation strategies [33]. It also helps to appreciate the extent to which the species genetic potential has been influenced by environmental gradients and geographical barriers to detect any developments in evolutionary processes such as speciation. Such investigations are also important for a highly synonymised species such as *O. lanceolata* to provide an understanding of evolutionary trends within the species over time [2]. For instance, analysis of spatial genetic structure helps to detect gene dispersal distance and the extent to which ecosystem disturbances influence the nonrandom distribution of genes in a population, leading to inbreeding and loss of genetic diversity [33].

Understanding the effect of anthropogenic bottlenecks and environmental gradients on species genetic diversity and structure is necessary to enhance conservation strategies for a species. Therefore, genetic data and information are needed to adequately understand what drives the species genetic adaptation potential and the present trends in genetic patterns to create informed conservation solutions. Due to its intense harvesting and ecological value in East Africa, conservation actions of *O. lanceolata* are urgent in the region. However, there is a lack of information on the species genetic patterns that can efficiently guide conservation measures. This study was aimed at strengthening the species conservation efforts in East Africa by determining the patterns in genetic diversity and structure and whether such patterns are influenced by geographical isolation and altitude gradient. Two specific objectives were addressed: (i) to characterise the genetic diversity and structure patterns of *O. lanceolata* across populations in Uganda and Kenya and (ii) to determine the relationship between geographical distance and genetic distance, as well as the effect of altitude on the species genetic structuring across its natural range in the two countries.

## Materials and methods

### Sampling

**Fig. 1:**
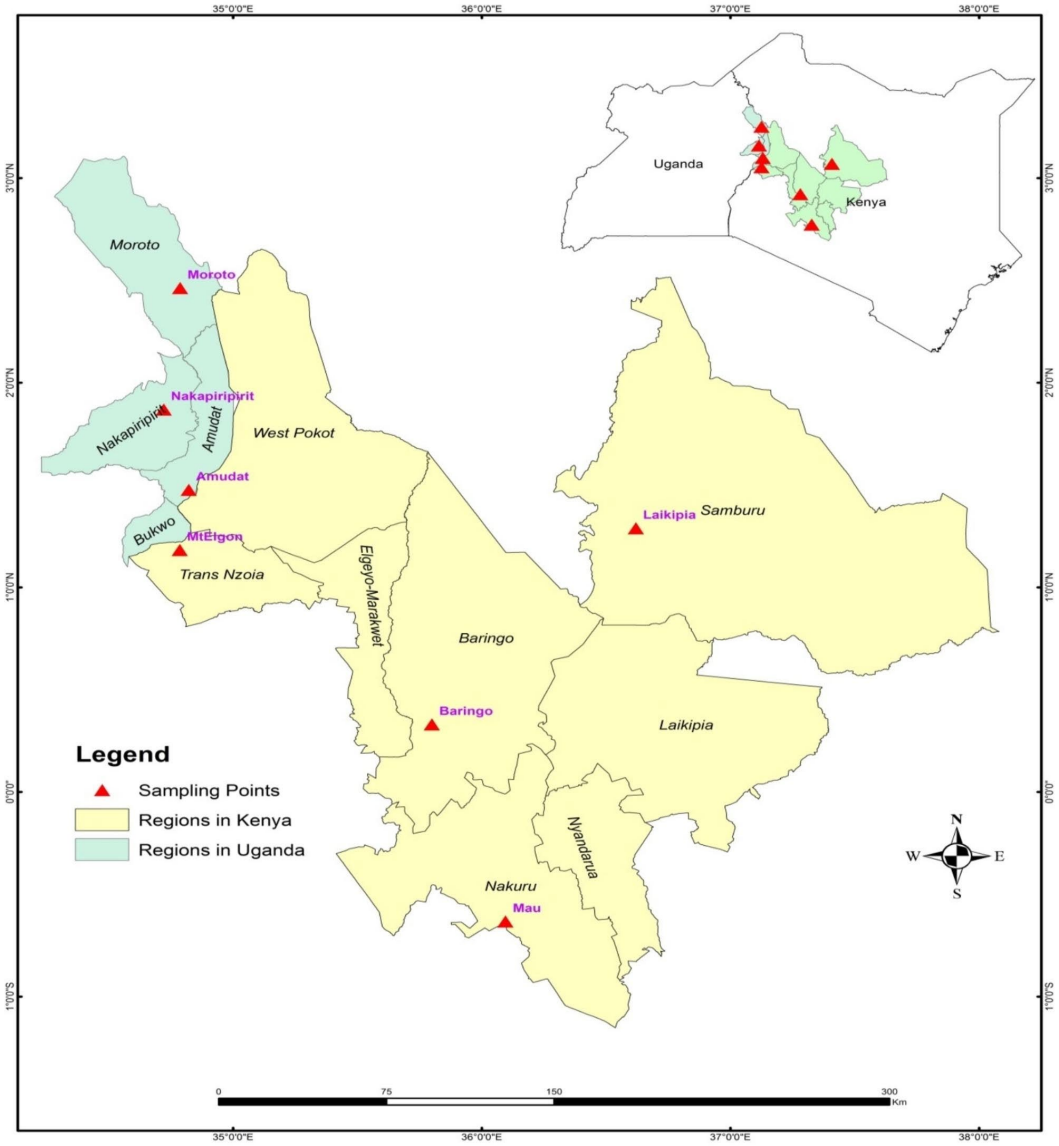
Geographic location of the 7 study populations of *O. lanceolata* in Uganda and Kenya. (Source: Open Street maps)

A total of seven populations, three from Uganda (Karamoja subregion) and four from Kenya in the rift-valley region, were sampled for genetic analysis (Fig. 1). The Karamoja populations were Amudat, Nakapiripirit and Moroto. The Kenyan populations included Mt. Elgon, Baringo, Laikipia and Mau. Leaf samples were collected from adult *Osyris lanceolata* trees using a pair of scissors, dried on silica gel in paper bags, and stored at room temperature until extraction of genomic DNA. A total of ninety-six (96) leaf samples from Uganda and one hundred fourteen (114) samples from Kenya were collected. At each sampling point of the *O. lanceolata* tree, fresh leaves were picked from each of the four directions; North – to southward, and East – to westwards. The sampling interval from one sampling point to another was not consistent due to the highly patchy distribution of the species, but the pattern followed the nearest neighbor approach, though not sampling aiming at collecting samples from the nearest individual, but a minimum distance of 20 m apart was maintained between sampling points. At each sampling site, coordinates were recorded by a GPS machine (Garmin). The voucher specimens of *O. lanceolata* genetic samples were wrapped in dry papers, well pressed and later deposited at Makerere University Herbarium, and Kenya Forestry Research Institute Herbarium (KEFRI) for further references. The list of herbarium accession numbers is provided (Appendix 1). The genetic analysis for all samples (Uganda and Kenya) was done at the Kenya Forestry Research Institute labs (KEFRI) at Muguga.

The detailed environmental variables of the sampled populations and their coordinates are indicated in Table 1.

**Table 1:** Sampling information of the seven populations of *Osyris lanceolata.* The annual rainfall and temperature provided were obtained from the respective weather stations for the sub-regions of Karamoja (Uganda) and Rift-valley (Kenya)

Population	Country	Sample Size (*n*)	Longitude	Latitude		Altitude (m)	Mean annual rainfall (mm)	Mean annual temperature (°C)
Mau	Kenya	30	36.08889	-0.607300		2288	1025.0	21.00
Laikipia	Kenya	26	36.37124	-0.117420		2055	207.27	15.70
Baringo	Kenya	30	35.78866	-0.396067		2040	635.00	25.00
Mt. Elgon	Kenya	28	34.81058	1.152700		2007	1280.0	18.50
Moroto	Uganda	20	34.759366	2.4524783		1754	800.00	20.60
Amudat	Uganda	60	34.80616	1.484444		1434	154.45	24.99
Nakapiripirit	Uganda	16	34.71965	1.869604		1401	156.53	25.33

### DNA extraction and PCR analysis

Total genomic DNA was extracted from dried leaf samples following the modified CTAB (acetyl-trimethyl-ammonium-bromide) protocol [34]. The DNA samples were quantified using a nanodrop spectrophotometer (BioSpec-nano, Shimadzu Biotech, Hamburg, Germany) to check the concentration and purity. After quantification, the samples were standardised to a working concentration of 10 ng/µl and stored at -20 °C until PCR analysis. Ten primer pairs developed by [23] for *O. lanceolata* were used to genetically characterise the seven populations in this study (Table 2). PCRs were conducted in a final volume of 5 µl using a Qiagen multiplex PCR kit (*Invitrogen, country*) following the manufacturer’s guidelines in a Veriti™ thermal cycler (Applied Biosystems). Each primer mix was composed of three to four markers each labelled with a different fluorescent dye (Table 2).

**Table 2:** *Osyris lanceolata* microsatellites and their allele sizes

Primer code	Direction	Primer sequence (5’to 3’)	Primer mixes	Allele sizes (bp)
KFOL2	F R	AGAATGTCATTTGAAGGCTCGA CCTTTCCTCCGTTCTCCTG	1	178–194
KFOL13	F R	TCCGAGGAACAGGGACTCTT AGCGAAGAACTCATGAGCGAA	1	139–165
KFOL17	F R	CATTGACGAATTGCATCCCGT CGTGAAGTTCAGTGCAAACC	1	178–220
KFOL24	F R	CAACTCGATCGTGCATTGGC TCCGCATATCCATTTGGCCG	2	219–263
KFOL28	F R	ATAAAGGCCCACGAGCTCAG AACATCGCCATGCAGAACAG	2	245–255
KFOL30	F R	CTAAACTGTCAGGGCTTGCT ATACCTTAGCTCCCGTTGCG	2	270–306
KFOL37	F R	TTTCTAGAGCTAACATACCTCTGAA ATGACCTGGGTGCTTTGCTG	3	300–340
KFOL42	F R	AGGTCCTCCTGAGAAT CATAGGGCTGTGATGCGTCA	3	315–337
KFOL47	F R	TTTGATCGTAAATTATAGATGTCCACA CCCTTGCTTGATCTCCAGGTA	3	353–387
KFOL48	F R	GAGTGCATGGAATTATGTGTGCGT TCGCCATGAGAAGGGTTACT	3	369–393

A touchdown thermocycling program used by [23] was followed in PCRs. This was programmed as follows: initial denaturation at 95 °C for 15 min, then 10 cycles at 94 °C for 30 s, 70 °C for 90 s, and 72 °C for 60 s. This was followed by 22 cycles at 94 °C for 30 s and 55 °C for 90 s. Annealing starts at 57^0^ C and decreases by 1^0^ C for each cycle and elongation at 72^0^ C for 1 min for 35 cycles. The final cycle was performed at 60 °C for 30 min. PCR analysis was performed using a Verit 96 Well Thermocycler (Applied Biosystems).

Fragment length analysis (FLA) was performed through capillary electrophoresis (CE) and scored against a 600 Liz internal size standard using a genetic analyser (3500, Applied Biosystems, HITACHI, Japan). Gene Mapper 5.0 software (Applied Biosystems, California, USA) was used to capture the genotypic data. To perform fragment analysis on a CE system, the fluorescently labelled primers were used to flank the *O. lanceolata* region or loci of interest and amplified by PCR before capillary electrophoresis. To prepare the CE, a special calibration with the corresponding matrix standard for the selected group of dyes was performed on the genetic analyser (Biosystems) to allow accurate detection of the dye-labelled primers. Also, each unknown sample was mixed with the size standard and formamide before CE to allow sizing of the sample peaks and correct for injection variations.

The data analysis software provides a profile of the separation, precisely calculates the sizes of the fragments, and determines the microsatellite alleles present in the sample. This is manifested in an electropherogram, which is a plot of DNA fragment sizes. The Fluorescently labelled fragments are separated by capillary electrophoresis and sized according to an internal standard. The peaks correspond to the different color dyes that are all resolvable and sized along the x-axis. The red line indicates low-level signals (noise) between the peaks.

## Data analyses

To prevent biases due to differences of sampling size between populations, a rarefaction approach was implemented for the calculation of population-based indices such as genetic diversity and differentiation estimates. This consisted of randomly subsampling 16 individuals (the lowest population size) for all populations. For the remaining analyses the whole data was used since these are individual based measures and the outcome is not affected by population sampling size.

GenAlEx software, version 6.51b2 [35], was used to assess the within- and among-population genetic diversity parameters, such as the percentage of polymorphic loci, observed heterozygosity (Ho), expected heterozygosity (He), Shannon information index (I), and genetic differentiation (F_ST_). Rarefied allelic richness (Ar) was calculated with the program HP-Rare [36] Deviations from Hardy-Weinberg equilibrium (HWE) and linkage disequilibrium among loci were tested using Gene pop software, version 4.7.5 [37]. The significance of deviations was computed using the Markov chain method (1000 iterations) [38]. Frequency of null alleles per marker and population was calculated with FreeNA [39]. Inbreeding coefficient (F_IS_) was calculated using FSTAT version 2.9.4 [40]. The possibility that genetic variation was affected by recent bottlenecks was tested using the software BOTTLENECK assuming the infinite allele model [41]. This program assumes that populations that underwent bottlenecks have a significantly higher expected heterozygosity than the expected under mutation-drift equilibrium. We tested if this difference was significantly larger than 0 using a one-tailed Wilcoxon test.

The population structural patterns were modelled using STRUCTURE software, version 4.5.7 [42]. The division between one to seven hypothetical populations (K = 1 to K = 10) was tested, and the best K value was evaluated by the deltaK criteria as implemented in Structure harvester [42]. STRUCTURE was run for 200,000 generations after a burn in period of 10,000 generations using the admixture ancestry model, correlated allele frequencies and not considering site locations as prior. A total of 10 replicates per K value were computed. The results per K value were summarised across replicates using the CLUMPAK server [43]. Pairwise F_ST_ was used as a measure of genetic differentiation between populations [44]. The partitioning of the genetic variation among populations and regions was analysed using analysis of molecular variance (AMOVA) in GenAlEx ver.5.1b2 [35]. The statistical testing for significance of AMOVA was determined by random permutations or shuffling of data 9999 times. The presence of isolation by distance, climatic variables (temperature and rainfall) and altitude was evaluated by testing for correlation between distance matrices of these parameters and pairwise F_ST_/(1-F_ST_) using mantel tests as implemented in the R package *ade4* [45]. The patterns of genetic structure among populations were visualised using principal coordinate analysis (PCoA). The existence of geographical barriers to gene-flow among populations was tested using the software Barrier v2.2 [46]. To this end, a pairwise distance matrix based on Nei DA was calculated with Population 1.2.31 [47]. Based on the Structure analysis up to four barriers were tested. Contemporary geneflow were calculated using the migration rate estimates calculated with BayesAss3 [48]. The program ran for 20 M generations sampling every 100th after a burn in of 10 M generations. Mixing parameters for allele frequency and inbreeding coefficient had to be set to 0.2.

## Results

### SSR markers, allele frequencies and Hardy-Weinberg equilibrium (HWE) tests

Overall, the markers produced 127 alleles, with 29.729 (SE = 1.582) as the mean number of alleles per locus. The primers KFOL17 (14 alleles) and KFOL24 (10 alleles) produced the highest number of alleles, while KFOL2 produced 4 alleles. The private alleles constituted approximately 22% (28 alleles) of the total alleles observed. All populations were highly polymorphic in a range of 80–100% polymorphism.

The patterns in allele frequencies varied across populations with some locus showing relatively consistent patterns between Kenya and Uganda irrespective of the altitude gradient while other alleles showed completely dissimilar frequencies along the altitude gradient (some alleles were aligned to either high or lower altitude populations in Uganda and Kenya. Another category of alleles showed dissimilar frequencies across the two countries. For instance, locus KFOL23 showed large disparity in frequency between lower altitude populations of Amudat and Nakapiripirit, and higher altitude populations of Moroto, and the rest of the Kenyan populations. Over 90% of alleles at this locus (KFOL23) were absent in Amudat and Nakapiripirit, but had almost uniform frequencies in Moroto and all the Kenyan populations which are higher altitude zones.

KFOL30 had a relatively uniform pattern in frequency across the Ugandan and Kenyan populations while locus KFOL37 had distinct patterns of allele frequencies clustered between low and high-altitude populations. For instance, Moroto and all Kenyan populations had similar pattern in allele frequency and richness while Nakapiripirit and Amudat also had similar pattern in allele frequency and richness. KFOL42 locus had allele frequencies and patterns in allele richness distributed or clustered along altitude gradient. KFOL 47 locus showed consistent pattern in allele frequency across all populations and altitudes. KFOL 48 was more characteristic in allele patterns along altitude gradient. Generally, the allele frequency patterns reflected presence of genetic differentiation along altitude gradients (low and high) and regions (Uganda and Kenya (Appendix 2).

Of the tests for deviation from HWE at each locus in each population, significant deviations were detected in 10 of the tests. The genetic marker KFOL47 showed the highest number of populations (85.71%) with significant deviations from HWE while KFOL37 had the least number of populations with significant deviations from HWE (28.57%) (Appendix 3, 4 and 5). In Kenya, Baringo showed 70% of the markers with significant deviations from HWE, followed by Mau and Laikipia, with a proportion (40%) of markers showing deviations from HWE. Mt. Elgon had the lowest proportion of markers (10%) showing deviations from HWE, indicating a population with the most stable allele frequencies. Mt. Elgon showed no significant deviations from linkage disequilibrium among loci, indicating random or independent association of alleles at different loci. Mau, Baringo, and Laikipia showed significant deviations from linkage disequilibrium among different loci. Among Ugandan populations, Moroto had 80% of the markers with significant deviations from HWE, while Nakapiripirit had the least number of markers (30%) with significant deviations (Appendix 3, 4 and 5). Overall, most Ugandan populations showed more markers deviating from HWE than the Kenyan populations. Nakapiripirit population showed the least number of deviations while Moroto and Amudat showing the highest numbers of deviations from linkage disequilibrium (LDE). Across populations, two markers (KFOL42 and KFOL47) showed higher frequency of null alleles (> 0.1) with Moroto the highest frequency of null alleles for most markers (Appendix 6).

#### Genetic diversity

There were distinct patterns in genetic diversity within and between populations across environmental gradients in Uganda and Kenya. These patterns were generally clustered in relation to altitude levels, and producing a dichotomy in genetic parameters among populations. Kenya populations were characteristic of higher altitude habitats than Ugandan populations in lower altitude areas. Across the two countries, mean allelic richness (Ar) was 3.985 and ranged from 3.9 to 5.87. The mean inbreeding coefficient was F_IS_ = 0.18, with a range of 0.060 to 0.410, indicating a deficit of heterozygosity. The mean expected heterozygosity was He = 0.606 ranging between 0.489 and 0.671, while the Shannon information index (I) ranged from 1.064 to 1.499, with a mean of 1.359 (Table 3).

Overall, the Kenyan populations showed higher genetic diversity than the Ugandan populations for all measures (Table 3). The average allelic richness in Uganda was lower (4.67) than Kenya (5.17). The same trend was observed for other genetic diversity measures. For example, average He in Uganda was 0.567 while in Kenya 0.635. Variation was also found within each country. In Uganda, the Moroto population had the highest genetic diversity (Ar = 5.41, I = 1.492, and He = 0.671), while Amudat had the lowest (Ar = 4.11, I = 1.064 He = 0.489). In Kenya, Baringo had a higher genetic diversity (Ar = 5.87, I = 1.499, He = 0.658), and Laikipia had the lowest (Ar = 3.9, I = 1.093, He = 0.573). The average inbreeding coefficient (F_IS_) values for Uganda populations were higher (F_IS_ = 0.2333) than those for Kenyan populations (F_IS_ = 0.138). Expected heterozygosity for two populations, Laikipia and Nakapiripirit, was significantly higher than the expected under the mutation-drift equilibrium indicating that these populations underwent a recent bottleneck (Table 3, Appendix 7).

**Table 3:** Mean genetic diversity indices over all loci across populations

Population	Ar	I	Ho	He	*F*_*IS*_	Bottleneck (*p*-value)
Mau	5.53	1.463	0.637	0.655	0.06	0.25
Laikipia	3.90	1.093	0.506	0.573	0.15	0.00
Baringo	5.87	1.499	0.508	0.658	0.26	0.65
Mt. Elgon	5.37	1.472	0.623	0.652	0.08	0.62
Moroto	5.41	1.492	0.415	0.671	0.41	0.05
Amudat	4.11	1.064	0.421	0.489	0.17	0.90
Nakapiripirit	4.50	1.171	0.491	0.541	0.12	0.00
Mean	4.96	1.322	0.414	0.606	0.18	

*Ar: Allelic richness I: information index, Ho: observed heterozygosity, He: expected heterozygosity, and F*_*IS*_: *fixation index*

#### Genetic structure and differentiation

The patterns of genetic divergence determined through AMOVA showed 91% of the divergence within individuals of *O. lanceolata* and 1% was among the populations (Table 4).

**Table 4:** AMOVA results and genetic structure among O. *lanceolata* populations

Source	Df	SS	MS	Est.var	(%)
Among regions (UG/KE)	1	79834.756	79834.756	281.235	3%
Among populations	5	98506.345	19701.269	77.535	1%
Among individuals	203	3136390.271	15450.198	7552.799	91%
Within individuals	210	72366.000	344.600	344.600	4%
Total	419	3387097.371		8256.168	100%

*Df = degree of freedom; SS = sum of squares; MS = mean squares; Est.var; estimated variance component;*

Based on F_ST_ values, there was little to moderate but significant genetic differentiation among most populations (Fsr = 0.118, p < 0.01), and among regions (Frt = 0.168, p < 0.01). However, great differentiation existed among individuals (Fst = 0.266, p < 0.01) (Appendix 7 and 8). The greatest interpopulation differentiation occurred between Mt. Elgon and Amudat (F_ST_ = 0.248). Among the Kenyan populations, great differentiation existed between Baringo and Laikipia, and the least genetic differentiation existed between Mt. Elgon and Baringo. Amudat and Moroto showed the greatest differentiation in Uganda, and the lowest occurred between Amudat and Nakapiripirit (Table 5 and Appendix 9).

**Table 5:** Pairwise population F_ST_ values for populations in Uganda and Kenya

	Mau	Laikipia	Baringo	Mt. Elgon	Moroto	Amudat	Nakapiripirit
Mau		0.001	0.002	0.002	0.001	0.001	0.001
Laikipia	0.079		0.001	0.001	0.001	0.001	0.001
Baringo	0.044	0.077		0.134	0.002	0.001	0.001
Mt. Elgon	0.035	0.070	0.024		0.001	0.001	0.001
Moroto	0.086	0.113	0.069	0.078		0.001	0.001
Amudat	0.237	0.247	0.210	0.234	0.159		0.002
Nakapiripirit	0.212	0.245	0.192	0.216	0.138	0.072	

*The FST values are shown below the diagonal while significance is shown in the upper panel*

**Fig. 2:**
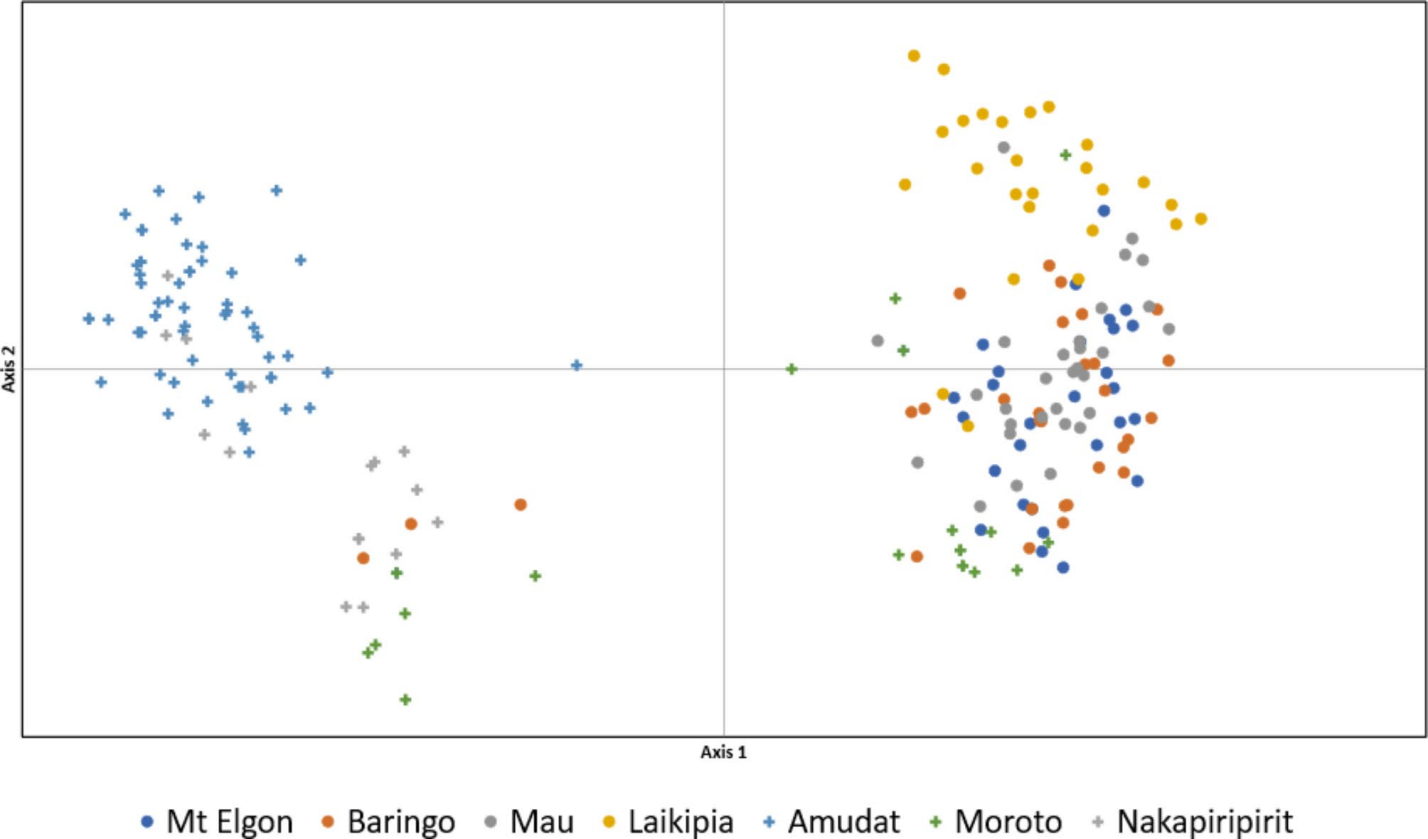
Principal coordinate analysis (PCoA) based on Nei‘s genetic distances for seven *O. lanceolata* populations

The principal coordinate analysis (PCoA) results showed two main clusters among the seven populations. The first two coordinates explained 33.76% of the total observed variation, suggesting the existence of distinct genetic structures among the populations (Fig. 2). The first coordinate separated mostly individuals from different regions/countries. The Moroto and Baringo populations had different individuals who clustered with populations of both countries with distinct clustering compared to other populations. Basically, the Amudat and Moroto populations form two clusters, with Nakapiripirit population spread out between the two. (Fig. 2).

**Fig. 3:**
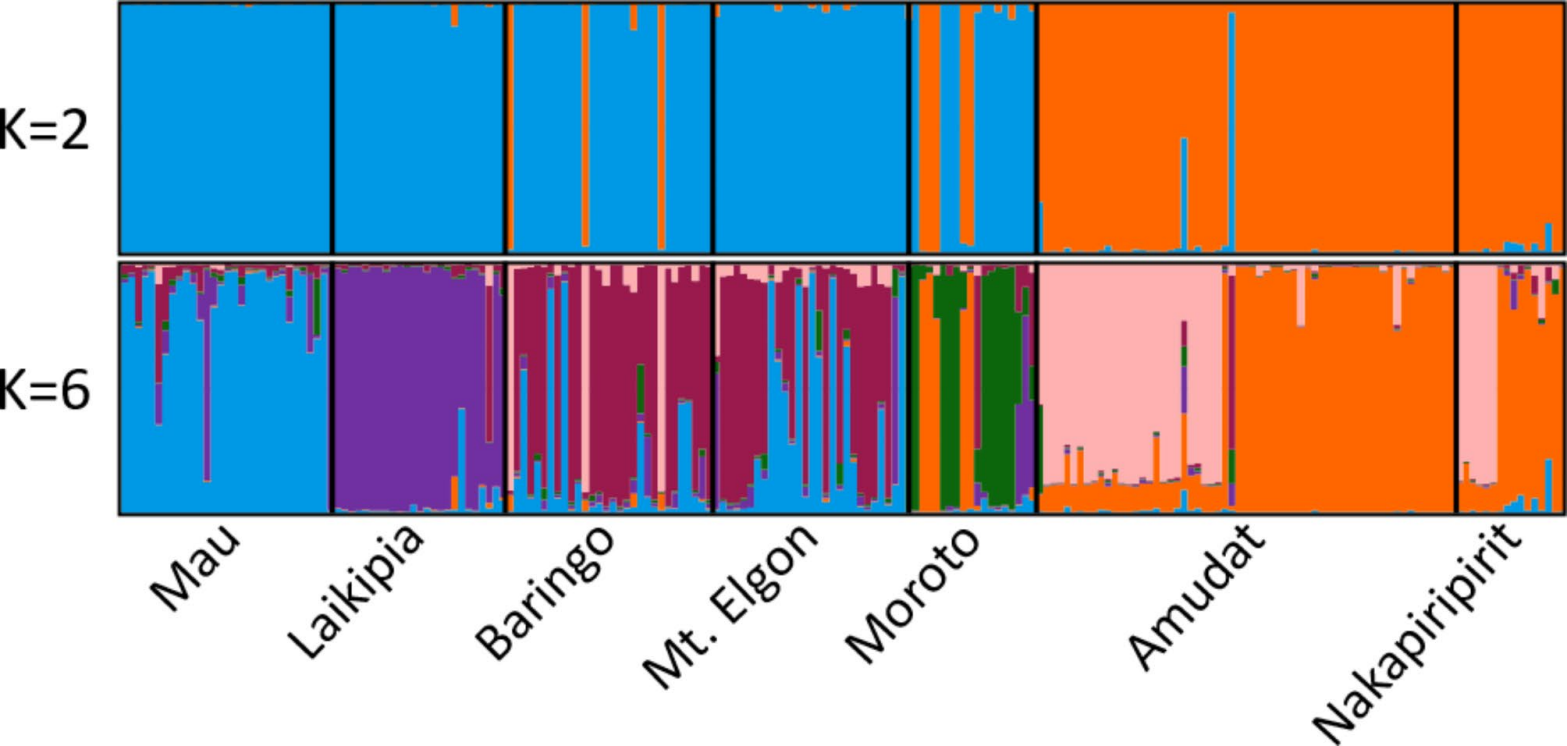
Bar plots of proportional group membership for the 210 trees genotyped at nuclear microsatellite loci for K = 2 (optimum) and 6 (the highest showing biological meaningful results). Each bar represents a single tree, with color representing the proportion of ancestry derived from each group. Black lines indicate the division between populations

Based on delta K variation, the best K value for the STRUCTURE analysis was two (K = 2) (Appendix 10 and 11), clustering together the populations from the same country. The main exception was Moroto, which showed assignment to both clusters with some degree of admixture. However, to a lower extent, some admixture was also observed in other populations, such as Amudat and Moroto. Some additional biologically meaningful clustering was found up to K = 6, but the greatest structuring occurred at K2 (Fig. 3). In summary, K = 6, shows that Laikipia and Mau are assigned to independent clusters; Baringo and Mt. Elgon share the same dominant cluster although with high degree of admixture with Mau and Amudat; individuals from Amudat and Nakapiripirit are assigned two clusters shared by both populations; and Moroto shows mixed assignment to the main Ugandan cluster and its own. The STRUCTURE analysis results are also consistent with the results from the PCoA (Fig. 2) as indicated in Fig. 3.

**Fig. 4:**
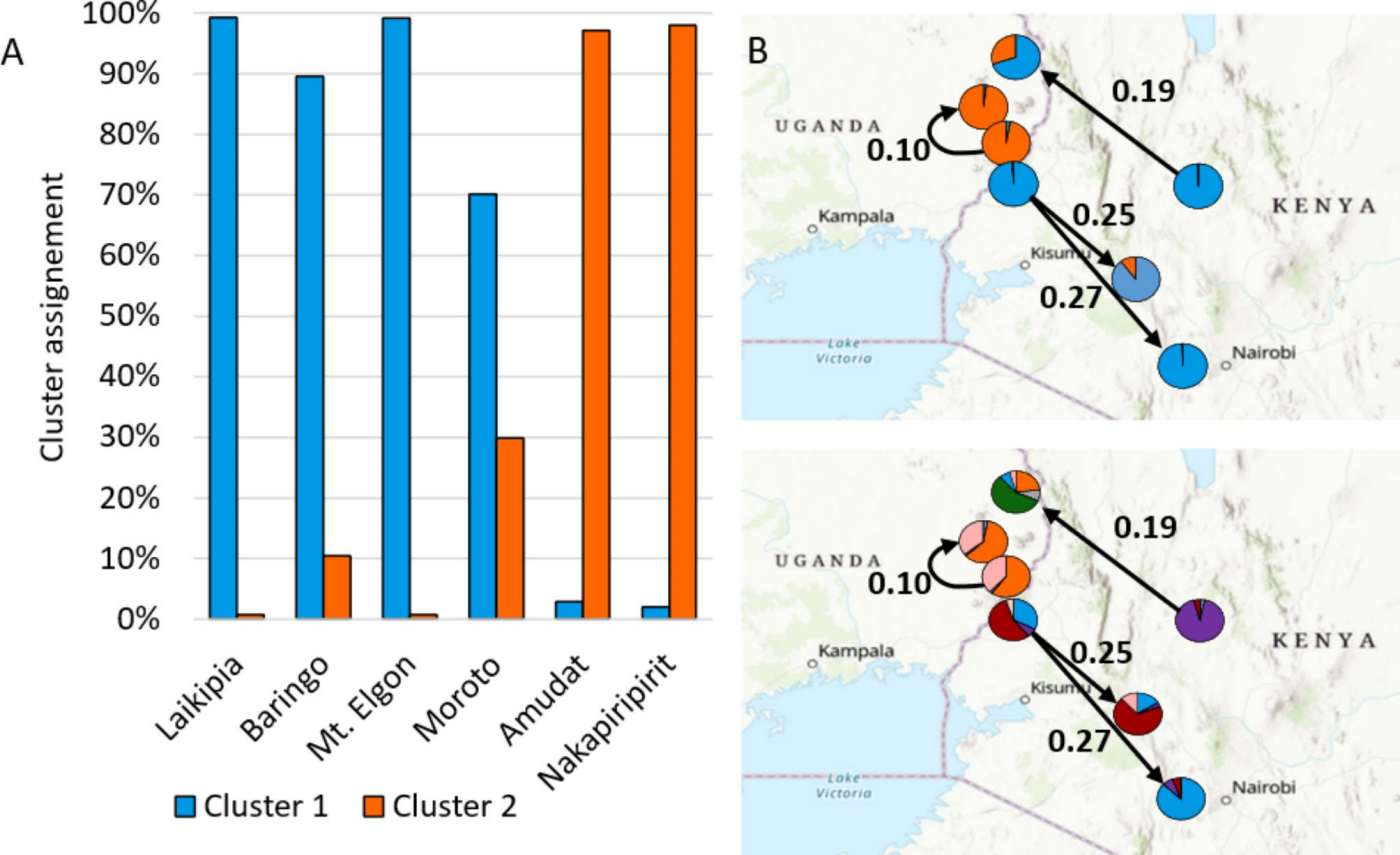
Distribution of genotypes in comparison to altitude and geographic location of population shown as cluster assignment in Structure. **A**, indicates the percentage of assignment at K = 2 sorted according to the altitude of the population location; **B** illustrates the proportion of Cluster assignment at K = 2 and K = 6 for the localities. (Source: GIS Open street map) and significant migration rates (arrows)

The distribution of genotypes among populations revealed the populations to have distinct genetic clusters assigned in relation to altitude. Some individuals of *O. lanceolata* species were observed to be characteristic of lower altitudes, and others, especially most of the Kenyan populations, were assigned to higher altitude habitats (Fig. 4).

The correlation between the pairwise F_ST_ matrix and the geographical distance for the pairwise comparisons among the 7 populations revealed no significant relationship (R^2^ = 0.057, p = 0.253). Similar patterns were revealed with temperature (R^2^ = 0.087, p = 0.111), rainfall (R^2^ = 0.039, p = 0.158). However, altitude revealed a strong and significant correlation with pairwise F_ST_ matrix (Appendix 12).

Possible geographical barriers identified were places in accordance with the Structure results (Appendix 13). The first barrier was placed between the Ugandan and Kenyan populations, which also corresponds to a difference of more than 500 m in altitude between Mt. Elgon and Amudat. The second barrier was placed between Moroto and the remaining Ugandan populations. This locality is also higher in altitude than the remaining two. The remaining two barriers were found between Kenyan populations isolating first Laikipia and second Mau from the remaining populations (Appendix 13).

We considered contemporary migration rates to be high when a proportion of migrants from a population originating from a second population were higher than 0.1. This was found to be the case for migrants found in the populations of: Baringo and Mau originating from Mt. Elgon, Moroto originating from Laikipia, and Nakapiripirit from Amudat (Fig. 4 and Appendix 14).

## Discussion

### Genetic diversity patterns

This is the first study to analyse the genetic diversity and structure of *O. lanceolata* in Africa using the species microsatellite markers (SSRs) developed in East Africa [23]. These markers were shown to be highly polymorphic and therefore informative for studying population genetic patterns of *O. lanceolata.* HWE deviations per marker across all populations may indicate genotyping errors due to null alleles and marker duplication. High prevalence of null alleles was found for two markers and some populations. Since null alleles are not equally distributed across the populations it is possible that some other biological factors are contributing to these deviations. These will be discussed below.

Based on the findings, the sampled populations showed relatively narrow ranges of genetic diversity (He = 0.49–0.68, Ar = 3.9–5.87). A higher divergence in genetic diversity was expected basing on the fact that the populations show clear genetic differentiation along divergent environmental gradients with different historical overexploitation regimes [49]. Similar levels of genetic diversity were found in other dioecious species such as *Mercurialis perennis* and several *Ficus* species, where genetic patterns are explained by stochastic events and sex ratios within the populations [50, 51]. Although the present study did not evaluate the impact of sex ratios on the genetic diversity of *O. lanceolata* and because the species is a dioecious plant [17], the sex ratio distribution could be an important factor influencing the observed variations in genetic diversities among and within the populations.

The findings pointing to higher genetic diversity in Kenyan populations than in Uganda are also consistent with earlier works reported by [23] and [24] despite the two studies using different markers. A study that used microsatellite markers [23] obtained higher genetic diversity (He) for the Mt. Elgon population (He = 0.000–0. 902) [23], while the use of ISSR markers also revealed higher genetic diversity for Mt. Elgon and Baringo populations [24]. Interestingly, most populations that showed higher genetic diversity (Baringo, Moroto, Mau and Mt. Elgon) were under protected status as government conservation areas, and all these populations occur in higher altitude areas (> 2000 m.a.s.l.) (Table 1). The higher degree of genetic diversity in these populations could be attributed to the effectiveness of conservation areas in preserving the germplasm of endangered species than communal and private habitats. Control measures, such as restricted resource harvesting and monitoring resource use, infer higher comparative advantages to species protection than communal populations. The higher genetic diversity across the four Kenyan populations could also be attributed to high levels of gene flow among populations [52], which is again supported by the fact that the highest migration rates were found between Kenyan populations. However, we cannot rule out the fact that SSR markers were developed for Kenyan *O. lanceolata* populations [23]. This might lead to ascertainment biases in genetic diversity estimates since the markers would preferably amplify Kenyan alleles [25, 49]. The high genetic diversity observed in Moroto may be a consequence of the high degree of admixture found in this population.

For the past ten years, *O. lanceolata* populations in Uganda and Kenya have experienced higher exploitation levels that have led to significant declines in species abundance, density [2, 12, 18] and changes in allele frequencies. Major disturbances included illegal destructive harvesting of *O. lanceolata* trees (uprooting whole plant – mainly female individuals), habitat clearing, destruction of host species for charcoal burning, timber, and fuel wood especially in Uganda [7, 12]. Such disturbances are expected to cause a decline in genetic diversity among individuals in the disturbed populations in addition to some populations being characteristic of lower altitude areas. Most Ugandan populations showed significant deviations from Hardy-Weinberg equilibrium in contrast to the Kenyan populations, which points to higher exposure to anthropogenic and ecological disturbances that might lead to significant loss of alleles from the population. The positive fixation index exhibited by all populations could be attributed to a lack of observed heterozygosity. Moroto and Amudat populations showed the highest number of markers deviating from HWE and the highest F values. Given that these populations showed substructure in both PCoA and STRUCTURE analysis, the higher deviations from HWE could be attributed to the Wahlund effect and not loss of genetic diversity. We found evidence of a recent bottleneck for the third population from Uganda, Nakapiripirit, which may explain the deviation from HWE. Interestingly, evidence of bottleneck was also found for the Kenyan population of Laikipia, the population with the least genetic diversity. The most likely explanation for these recent bottlenecks is overexploitation of *O. lanceolata* in both countries contributing to the loss of genetic diversity. At long term this might have particularly drastic consequences to the species populations in Uganda where the control measures for harvesting of *O. lanceolata* resources are limited.

The overexploitation of *O. lanceolata* was detected earlier in Kenya and measures were established in 2007 to protect the resource base through a presidential decree that banned harvesting and trade in *O. lanceolata* resources [3]. Most likely, the strict control of *O. lanceolata* resource utilisation in Kenya shifted pressure to Ugandan populations to meet the illegal market demand for the species resources [12, 18]. Unfortunately, the lack of quick deterrent measures to curb the destructive harvesting of the species in Uganda could have severely led to loss of important alleles from the populations. The destructive harvesting of the species (uprooting of the whole plant) for a longer period of time could have played an important role in changing the genetic diversity levels in terms of allele frequencies among the Uganda populations, hence weakening the species genetic potential. Also, the level of genetic drift among and within *O. lanceolata* individuals could have been higher in community habitats compared to habitats in protected areas such as Mt. Elgon in Kenya and Mt. Moroto and this supported by the lower genetic diversity in populations on communal habitats such as Nakapiripirit and Amudat than those in protected areas of Mt. Moroto which may explain the differing patterns in genetic diversity and deviations from HWE in these populations.

### Genetic structure patterns

The seven populations were mainly structured into two major genetic clusters in relation to altitude gradients and some populations showed genetic admixture (Baringo and Moroto) between Uganda and Kenya. This was further supported by the PCoA clustering pattern along the first coordinate and high F_ST_ values between some populations of different countries, and the fact that AMOVA showed that groupings based on country of origin explained a higher proportion of the variation than those based on locality. The genetic structuring between Kenya and Uganda *O. lanceolata* populations could be attributed to ecological, evolutionary, and anthropogenic factors.

The presence of geographical barriers and environmental gradients, such as differences in altitude, also influence genetic differentiation through the isolation of populations [53]. Geographically, the populations could be spatially isolated to the extent that dispersal mechanisms cannot facilitate free interbreeding between *O. lanceolata* individuals in the two regions. Second, the *O. lanceolata* habitats in Uganda and Kenya are highly fragmented by geographical barriers, leading to continued inbreeding that is reflected in the high levels of fixation indices among the Kenyan and Ugandan populations. For instance, Mt. Kadam separates Nakapiripirit population from Amudat, Mt. Moroto, separates Moroto populations from Amudat and Nakapiripirit, and the long distance between Moroto and other populations in Kenya such as Baringo, Mau and Laikipia are barriers that obstruct continuous dispersal and flow of genes among the populations, hence leading to gradual differentiation, in absence of long-distance dispersal and geneflow. In addition, most Ugandan populations (Amudat and Nakapiripirit) occurred in lower altitude areas (≤ 1500 m.a.s.l.) compared to Moroto (≥ 1700 m.a.s.l.), and Kenyan populations found at higher altitudes > 2000 m.a.s.l.) (Table 1). The difference in altitude creates variations in climatic parameters such as rainfall and temperatures (Table 1), which impact on the species’ eco-physiological and genetic adaptation potential to prevailing conditions.

The results obtained from the barrier analysis support the hypothesis that altitude plays an important role in restricting geneflow since the main barriers were places between populations having the highest altitudinal differences (Appendix 13). Moreover, genetic distance was highly correlated with altitudinal distances. These variations in environmental gradients, such as altitude coupled with geographic barriers that obstruct gene flow and dispersal may eventually lead to microevolution and allopatric speciation. Therefore, there is a possibility that sampled populations correspond to different subspecies of *O. lanceolata* that are gradually emerging out of *O. lanceolata* taxon due to environmental isolation but not yet recognised. Such subspecies could contribute to the observed differences and patterns in the genetic diversity and structuring. This hypothesis needs to be tested with phylogenetic approaches using sequencing markers and supported by morphological data. An earlier study to understand the evidence of gene flow and selection in *Santalum alba* in different populations observed that natural barriers influence the fragmentation of habitats, which eventually disrupts gene flow among populations [54]. Another recent study also revealed that altitude has a significant effect on the morphology of *O. lanceolata* and is one of the drivers to the species’ morphological plasticity [12]. Additionally, the high level of anthropogenic disturbances exposed to the species habitats, including destructive harvesting of the species between Uganda and Kenya populations, have gradually contributed to significant variations in allele frequencies and genetic variations among populations, hence leading to genetic structuring. There are reports of illegal species exploitation in Moroto, Nakapiripirit, Amudat and among Kenyan populations before regulations were established by the Kenyan government [3, 14, 16].

Nevertheless, the data supports the existence of long-distance dispersal as it is shown by relatively high migration rate between Laikipia and Moroto (Appendix 14), which can also explain the admixture pattern found in the latter. Long distance gene-flow has been observed among populations of *Acacia senegal* [55] and *Santalum alba* [54]. In relation to populations in this study, long-distance gene flow could result from deliberate transfer of *O. lanceolata* planting materials such as seeds and seedlings from Uganda to Kenya and Kenya to Uganda, hence causing admixture of genes between countries through genetic translocations. Thus, the genetic admixture could be attributed to the occurrence of long-distance dispersal or human-mediated translocation between isolated populations, leading to the introduction of new genes from distant populations. Additionally, natural long-distance dispersal is possible through seed dispersal agents such as birds and mammals [55]. Natural admixture can be beneficial since it increases standing variation and forms new genotypes that can be useful in genetic improvement programs [54]. On the other hand, artificial admixture has been shown to break local adaptation and contribute to outbreeding depression [54]. Thus, it is important to further explore the processes contributing to the admixture found in some of these populations, its effects on local populations, and the role of such processes in the conservation of *O. lanceolata* genetic resources.

The underlying pattern of genetic structure observed for the populations indicates the possibility of not yet recognised taxonomic units in the study populations. If that is the case, the split of *O. lanceolata* populations into two larger clusters and the assignment of some individuals within one population to two clusters point to the existence of one subspecies with a more westerly, low elevation and another, including most populations in Kenya, with an easterly, high elevation distribution. As we reviewed recently, *O. lanceolata* has a confusing taxonomy with extensive synonymisation and high morphological variation across a very wide distribution range [2]. The concept of treating all these individuals as one species might be unsuitable, which might also include the possibility of smaller range species differentiation. This would also have implications in the interpretations of our results. For example, Moroto would be a contact point between these distinct taxa where they can interbreed.

This topic will be one focus of our future investigations considering sequence-based chloroplast markers. It is important to note that management would be significantly impacted by the existence of cryptic subspecies. The correlation of structure with altitude indicates presence of ecological differentiation within the *O. lanceolata* taxon that needs to be considered in management strategies. This is because, the definition of a management unit would therefore be more difficult, when highlighting the necessity of considering the suitable provenances to provide material for ex situ propagation and cultivation of *O. lanceolata* species.

### Implications for *O. lanceolata* conservation

This study highlights areas with implications for the conservation of genetic resources of *O. lanceolata* in East Africa. The species genetic structure is clearly organised into two genetic units that should be conserved from extreme population disturbances. The presence of significant levels of differentiation and structuring among populations in Kenya and Uganda requires strategic interventions to prevent adverse effects from continuous population differentiation, such as genetic diversity loss, reduction in population size, isolation and loss of the species’ ability to adapt and survive.

Although Ugandan populations showed slightly lower genetic diversity than most Kenyan populations, there are populations with higher genetic diversity such as Moroto that could serve as suitable provenances for boosting in situ and ex situ conservation programs. However, a better criterion for identifying suitable provenances should take into consideration the true taxonomic identity, morphological traits, and ecological, genetic, and biochemical properties of the target populations to inform credible management decisions that will promote the conservation of desirable traits for the species. The two main genetic groups found are associated with altitude variations that might translate in different adaptive traits. Thus, ex-situ activities should consider the divergent adaptive potential of these two units that might have a maladaptive nature if translocated into non optimal conditions. In this scope Moroto, might be an important population to study since both units seem to coexist.

It is also important to evaluate if the genetic groups found show different biochemical properties such as oil yield, composition, and quality among the populations in East Africa. By identifying genotypes that are more fit to be cultivated, one can more efficiently pick germplasm for selective breeding. This would release the pressure from harvesting natural populations and hence guarantee long-term conservation and sustainable use of the species.

Finally, the findings inferred evolutionary, anthropogenic and ecophysiological impacts on population genetic diversity and structure patterns of *Osyris lanceolata* in Uganda and Kenya. In fact, two populations were identified to have undergone a recent bottleneck and might require a more urgent conservation action. The high genetic divergence between some Ugandan and Kenyan populations should also be considered an opportunity for exploring further speciation within *O. lanceolata* and developing strategies to conserve a wide range of genetically diverse species (*O. lanceolata*) with a higher genetic potential for adaptation in a wide range of semiarid habitats in Africa and beyond.

## Conclusions

This study investigated the genetic diversity and structure patterns of *O. lanceolata* across seven populations in Uganda and Kenya. There was evidence that Ugandan populations were more threatened than Kenyan populations due to genetic diversity loss. Population bottlenecks, evolutionary processes within the species, and environmental gradients might be key drivers of species genetic structuring between Uganda and Kenya rather than isolation by distance. The observed evidence of genetic admixture and the causes and consequences of species fitness should be further investigated. Additionally, the large genetic divergence between *O. lanceolata* populations in both countries should present an opportunity for conservation of a wide range of the species gene pool. These results are important to guide conservation efforts. For example, priority in restoration efforts might be given to populations that have experienced recent bottlenecks, and ex-situ programs should consider the different genetic units found and their potentially divergent adaptive ability.

### Electronic supplementary material

Below is the link to the electronic supplementary material.

Supplementary Materials (Appendices): Appendix 1: Table 1: Herbarium accession numbers and voucher specimens for Osyris lanceolata; Appendix 2: Patterns of allele frequencies among sampled populations in Uganda and Kenya; Appendix 3 Table 3: Probability values for Hardy Weinberg equilibrium (HWE) tests in populations; Appendix 4: Table 4 p values for genotypic linkage disequilibrium for each pair of loci across all populations; Appendix 5 Table 5 Chi-square tests for Hardy-Weinberg equilibrium per loci per population; Appendix 6 Table 6: Null alleles estimation among populations; Appendix 7: Table 7: Genetic diversity indices for the 10 loci over the seven studied populations; Appendix 8: Table 8 F statistics results; Appendix 9 Table 9: Pairwise FST and Nm values across populations in Uganda and Kenya; Appendix 10: Table 10 Delta K values for the seven K populations proposed; Appendix 11 Criteria for detecting the optimum K values by Delta K values; Appendix 12: Results of Mantel Tests for isolation by distance, temperature, rainfall and altitude; Appendix 13: Barriers identified among sampled populations; Appendix 14: Migration rates across sink and source populations in Uganda and Kenya

## Data Availability

The data supporting the conclusions of this study are within the manuscript. The dataset that was used or analysed during this study are available from the corresponding author on reasonable request.

## References

[1] Mwangi JG, Haggar J, Mohammed S, Santika T, Umar KM. (2023). The ecology, distribution, and anthropogenic threats of multipurpose hemi-parasitic plant Osyris lanceolata. J Nat Conserv, 126478.

[2] Mugula BB, Kiboi SK, Kanya IJ, Egeru A, Okullo P, Curto M, Meimberg H. (2021) Knowledge gaps in taxonomy, ecology, population distribution drivers and genetic diversity of African sandalwood (*Osyris lanceolata* Hochst. &Steud.): A scoping review for conservation, Plants, 10(9). Available at: 10.3390/plants10091780. 10.3390/plants10091780 PMC8465005 34579313

[3] CITES.2013 (2013). Consideration of proposals for amendment of appendices I and II.

[4] Teixeira da Silva JA. (2016). Sandalwood spike disease: a brief synthesis. Environmental and Experimental Biology, 14(4), pp. 199–204. Available at: 10.22364/eeb.14.26.

[5] Teklehaimanot Z, Mwang’ingo PL, Mugasha AG, Ruffo CK (2004). Influence of the origin of stem cutting, season of collection and auxin application on the vegetative propagation of African sandalwood (*Osyris lanceolata*) in Tanzania. South Afr Forestry J.

[6] Njoroge GN, Bussmann RW. (2006) Diversity and utilisation of antimalarial ethnophytotherapeutic remedies among the Kikuyus (Central Kenya), J. Ethnobiol. Ethnomed, 2(8). 10.1186/1746-4269-2-8 PMC1397805 16451716

[7] Ochanda KV. (2009) Conservation and Management of Sandalwood Trees: (Osyris lanceolata Hochst & Steudel) in Chyullu Hills Kibwezi District, Kenya. MSc. Thesis, Kenyatta University, Nairobi, Kenya.

[8] Orwa C, Mutua A, Kindt R, Jamnadass R, Simons A (2009). Agroforestree Database: a Tree reference and selection guide.

[9] Singh H, Kumar S, Arya A (2023). Ethno-dermatological relevance of medicinal plants from the Indian himalayan region and its implications on cosmeceuticals: a review. J Drug Res Ayurvedic Sci.

[10] Xiaohai LIU, Yuntao GA, O, Khan S, Gang D, Aikui C, Li L, Xuecan WU (2008). Accumulation of Pb, Cu, and Zn in native plants growing on contaminated sites and their potential accumulation capacity in Heqing, Yunnan. J Environ Sci.

[11] Malabadi RB, Kolkar KP, Chalannavar RK, Munhoz ANR, Abdi G, Baijnath H (2023). Cannabis sativa: Dioecious into Monoecious plants influencing sex determination. Int J Res Innovations Appl Sci (IJRIAS).

[12] Mugula et al., Characterisation of the population structure and distribution drivers of *O. lanceolata* in the Karamoja subregion, Uganda, (In Press).

[13] Page T, Hannington T, Bunt C, Potrawiak A, Berry A. (2012) Opportunities for the Smallholder Sandalwood Industry in Vanuatu; ACIAR Technical Reports No. 79; Australian Centre for International Agricultural Research: Canberra, Australia.

[14] Bunei EK. 2017. The hunt for the precious wood. Society and Business Review, 12(1), pp. 63–76. Available at: 10.1108/sbr-04-2016-0025.

[15] Kioko EM, KINYANJUI MM. Commodifying East Africa’s sandalwood: Organised crime and community participation in the transnational smuggling of an endangered plant. *Commodifying the ‘wild’: Conservation, markets and the environment in southern and eastern Africa*.

[16] Tajuba P. How Oil Firm Is Burning up Karamoja Valuable Trees. Available online: http://www.monitor.co.ug/artsculture/Reviews/oil-firm-burning-up-Karamoja-valuable-trees/691232-2996318-r5xq40/index.html (accessed on 6 August 2021).

[17] Mwang’ingo PL, Teklehaimanot Z, Hall JB, Zilihona JE (2007). Sex distribution, reproductive biology and regeneration in the dioecious species *Osyris lanceolata* (African sandalwood) in Tanzania. Tanzan J Forestry Nat Conserv.

[18] Díaz-Barradas MC, Valera J, Esquivias MP, Zunzunegui M (2023). The hemiparasitic shrub *Osyris lanceolata* (Santalaceae) does not disturb the ecophysiology of its hosts. Flora.

[19] Gebirehiwot HT, Kedanu AA, Guangul AA, Adugna MT (2023). Floristic Composition, structure, and regeneration status of Woody Plant species in Hurubu Natural Forest, North Shewa, Oromia Region, Ethiopia. J Landsc Ecol.

[20] Giathi G, Kamondo BM, Njuguna JW, Mwangi S, Kipkoech N, Ingutia C (2023). Rooting African sandalwood stem cuttings using low-cost technology employed in the commercial propagation of Camellia sinensis in Kenya. J Hortic Forestry.

[21] Andiego PK (2022). Phenology and reproductive biology of Kenyan endangered sandalwood Osyris lanceolata Hochst. & Steud. J Environ Stud.

[22] Mwaura A, Kamau J, Ombori O. (2020). An ethnobotanical study of medicinal plants commonly traded in Kajiado, Narok and Nairobi counties, Kenya: Medicinal Plant species Traded in Kenya. East Afr J Sci Technol Innov, 1(3).

[23] Otieno JO, Omondi SF, Perry A, Odee DW, Makatiani ET, Kiplagat O, Cavers S (2016). Development and characterisation of microsatellite markers for *Osyris Lanceolata* Hochst. & Steud., an endangered African 1279 sandalwood tree species. Trop. Plant Res.

[24] Andiego KP, Dangasuk OG, Odee DW, Omondi FS, Otieno DF, Balozi BK (2019). Genetic diversity of endangered sandalwood (Osyris lanceolata) populations in Kenya using ISSR molecular markers. East Afr Agricultural Forestry J.

[25] Curto M, Beja A, Nogueira P, Beja M, P., Amorim F. (2015). Influence of past agricultural fragmentation to the genetic structure of *Juniperus oxycedrus* in a Mediterranean landscape, (April). *Tree Genetics & Genomes (2015) 11:32*10.1007/s11295-015-0861-2.

[26] Ellegren H, Galtier N. (2016) Determinants of genetic diversity. Nat. Rev. Genet. 2016, 17, 422–433, 10.1038/nrg.2016.58. 10.1038/nrg.2016.58 27265362

[27] Fuentes-pardo AP, Ruzzante DE. (2017). Whole-genome sequencing approaches for conservation biology: Advantages, limitations and practical recommendations, (June). 10.1111/mec.14264. 10.1111/mec.14264 28746784

[28] Alfaro RI, Fady B, Vendramin GG, Dawson IK, Fleming RA, Sáenz-Romero C, Loo J (2014). The role of forest genetic resources in responding to biotic and abiotic factors in the context of anthropogenic climate change. For Ecol Manag.

[29] Graudal, L., Aravanopoulos, F., Bennadji, Z., Changtragoon, S., Fady, B., Kjær, E.D., … Vendramin, G. G. (2014). Global to local genetic diversity indicators of evolutionary potential in tree species within and outside forests. *Forest Ecology and Management*, 333, 35–51. https://doi.org/10.1016/j.foreco.2014.05.002.

[30] Farwig N, Braun ÆC, Bo ÆK. (2008). Human disturbance reduces genetic diversity of an endangered tropical tree, Prunus africana (Rosacea), 317–26. 10.1007/s10592-007-9343-x.

[31] De Barba M, Miquel C, Lobréaux S, Quenette PY, Swenson JE, Taberlet P (2017). High-throughput microsatellite genotyping in ecology: improved accuracy, efficiency, standardisation and success with low-quantity and degraded DNA. Mol Ecol Resour.

[32] Vartia S, Villanueva-Cañas JL, Finarelli J, Farrell ED, Collins PC, Hughes G, Carlsson JEL, Gauthier DT, McGinnity P, Cross TF (2016). A novel method of microsatellite genotyping-by-sequencing using individual combinatorial barcoding. R Soc Open Sci.

[33] Zong JW, Zhao TT, Ma QH, Liang LS, Wang GX (2015). Assessment of Genetic Diversity and Population Genetic structure of *Corylusmand Shurica* in China using SSR markers. PLoS ONE.

[34] Hanaoka S, Omondi S, Machua J. (2013). Basic molecular techniques for tree breeding—experimental protocols. *Tokyo, Japan: Sankeisha*.

[35] Peakall R, Smouse PE (2006). GenAlEx 6: genetic analysis in Excel. Population genetic software for teaching and research. Mol Ecol Notes.

[36] Kalinowski ST (2005). HP-Rare: a computer program for performing rarefaction on measures of allelic diversity. Mol Ecol Notes.

[37] Glennon KL, Roux L, J. J., Thompson DI. (2023). Genetic insights into pepper-bark tree (*Warburgia Salutaris*) reproduction in South Africa. Conserv Genet, 1–9.

[38] Tang, M., Liu, L., Hu, X., Zheng, H., Wang, Z., Liu, Y., … Xie, S. (2023). Genome-wide characterization of R2R3-MYB gene family in Santalum album and their expression analysis under cold stress. *Frontiers in Plant Science*, *14*, 1142562. 10.3389/fpls.2023.1142562 PMC10017448 36938022

[39] Chapuis M-P, Lecoq M, Michalakis Y, Loiseau A, Sword GA, Piry S, Estoup A (2008). Do outbreaks affect genetic population structure? A worldwide survey in Locusta Migratoria, a pest plagued by microsatellite null alleles. Mol Ecol.

[40] Duc, N. M., Hoang, N. H., Giang, T. T. H., Huong, N. T. T., Duy, V. D., Hong, N. P.L., … Tam, N. M. (2023). Genetic Variation and Evolutionary History of the Threatened Dipterocarpus turbinatus CF Gaertn. Detected Using Microsatellites. *Diversity*, *15*(8), 894.

[41] Piry S, Luikart G, Cornuet JM (1999). Computer note. BOTTLENECK: a computer program for detecting recent reductions in the effective size using allele frequency data. J Hered.

[42] Earl DA, VonHoldt BM (2012). STRUCTURE HARVESTER: a website and program for visualising STRUCTURE output and implementing the Evanno method. Conserv Genet Resour.

[43] Kopelman NM, Mayzel J, Jakobsson M, Rosenberg NA, Mayrose I (2015). Clumpak: a program for identifying clustering modes and packaging population structure inferences across K. Mol Ecol Resour.

[44] Ma L, Ji YJ, Zhang DX (2015). Statistical measures of genetic differentiation of populations: Rationales, history and current states. Curr Zool.

[45] Dray S, Dufour AB (2007). The ade4 package: implementing the duality diagram for ecologists. J Stat Softw.

[46] Manni FE, Guérard, Heyer E (2004). Geographic patterns of (genetic, morphologic, linguistic) variation: how barriers can be detected by Monmonier’s algorithm. Hum Biol.

[47] Langella O. 1999. Populations 1.2.31. http://bioinformatics.org/~tryphon/populations/.47.

[48] Wilson GA, Rannala B (2003). Bayesian inference of recent migration rates using multilocus genotypes. Genetics.

[49] Heslot N, Rutkoski J, Poland J, Jannink JL, Sorrells ME. (2013). Impact of marker ascertainment bias on genomic selection accuracy and estimates of genetic diversity. PLoS ONE, *8*(9), e74612.52. 10.1371/journal.pone.0074612 PMC3764096 24040295

[50] Vandepitte K, Honnay O, De Meyer T, Jacquemyn H, Roldán-Ruiz I (2010). Patterns of sex ratio variation and genetic diversity in the dioecious forest perennial Mercurialis perennis. Plant Ecol.

[51] Nazareno AG, Alzate-Marin AL, Pereira RAS (2013). Dioecy, more than monoecy, affects plant spatial genetic structure: the case study of Ficus. Ecol Evol.

[52] Muriira NG, Muchugi A, Yu A, Xu J, Liu A (2018). Genetic diversity analysis reveals genetic differentiation and strong population structure in Calotropis plants. Sci Rep.

[53] Liu Y, Dietrich CH, Wei C (2019). Genetic divergence, population differentiation and phylogeography of the cicada *Subpsaltria Yangi* based on molecular and acoustic data: an example of the early stage of speciation. BMC Evol Biol.

[54] Ratnaningrum YW, Indrioko S, Faridah E, Syahbudin A (2017). Gene flow and selection evidence of sandalwood (Santalum album) under various population structures in Gunung Sewu (Java, Indonesia), and its effects on genetic differentiation. Biodiversitas J Biol Divers.

[55] Omondi SF, Githae EW, Khasa DP. (2023). Long-distance gene flow in Acacia senegal: hope for disturbed and fragmented populations. Ecol Evol, *13*(7), e10292.55. 10.1002/ece3.10292 PMC10337015 37449018

