# Supplementary material for: Microsatellites reveal divergence in population genetic diversity, and structure of osyris lanceolata (santalaceae) in Uganda and Kenya
Source: BMC Ecol Evol. 2023 Dec 8;23:73. doi: 10.1186/s12862-023-02182-2 (PMC10704637; doi:10.1186/s12862-023-02182-2)
Supplement: Supplementary file 1 — Supplementary Materials (Appendices): Appendix 1: Table 1: Herbarium accession numbers and voucher specimens for Osyris lanceolata; Appendix 2: Patterns of allele frequencies among sampled populations in Uganda and Kenya; Appendix 3 Table 3: Probability values for Hardy Weinberg equilibrium (HWE) tests in populations; Appendix 4: Table 4 p values for genotypic linkage disequilibrium for each pair of loci across all populations; Appendix 5 Table 5 Chi-square tests for Hardy-Weinberg equilibrium per loci per population; Appendix 6 Table 6: Null alleles estimation among populations; Appendix 7: Table 7: Genetic diversity indices for the 10 loci over the seven studied populations; Appendix 8: Table 8 F statistics results; Appendix 9 Table 9: Pairwise FST and Nm values across populations in Uganda and Kenya; Appendix 10: Table 10 Delta K values for the seven K populations proposed; Appendix 11 Criteria for detecting the optimum K values by Delta K values; Appendix 12: Results of Mantel Tests for isolation by distance, temperature, rainfall and altitude; Appendix 13: Barriers identified among sampled populations; Appendix 14: Migration rates across sink and source populations in Uganda and Kenya [file 12862_2023_2182_MOESM1_ESM.doc]

Supplementary materials (Appendices)

# Appendix 1: Table 1: Herbarium accession numbers and voucher specimens for *Osyris lanceolata*

| Specimen Voucher number | Accession numbers | Herbarium |
| --- | --- | --- |
| 45 | 51062 | Makerere University |
| 33 | 51063 | Makerere University |
| 5 | 51064 | Makerere University |
| 75 | 51065 | Makerere University |
| 65 | 51066 | Makerere University |
| 76 | 51067 | Makerere University |
| 68 | 51068 | Makerere University |
| 69 | 51069 | Makerere University |
| 29 | 51070 | Makerere University |
| 70 | 51079 | Makerere University |
| 71 | 51080 | Makerere University |
| 9 | 51081 | Makerere University |
| 60 | 51082 | Makerere University |
| 31 | 51083 | Makerere University |
| 6 | 51084 | Makerere University |
| 15 | 51096 | Makerere University |
| 180 | 63843 | Kenya Forestry Research Institute Herbarium |
| 197 | 63911 | Kenya Forestry Research Institute Herbarium |
| 199 | 63913 | Kenya Forestry Research Institute Herbarium |
| 232 | 63934 | Kenya Forestry Research Institute Herbarium |

**Appendix 2 Patterns of allele frequencies among sampled populations in Uganda and Kenya**


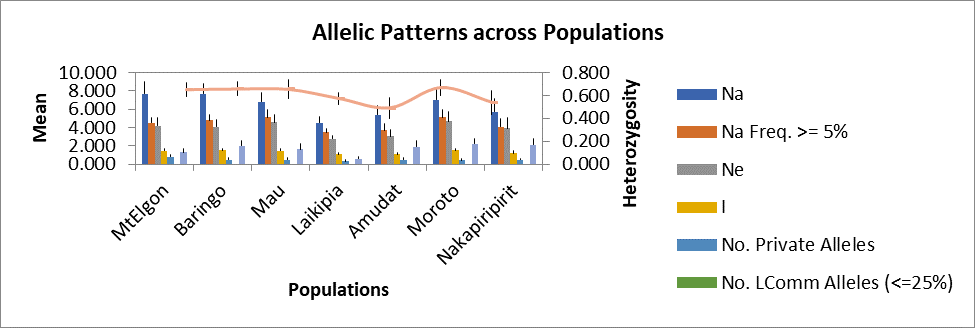


Figure 2 Allelic patterns of sampled populations in Uganda and Kenya

**Appendix 3 Table 3: Probability values for Hardy Weinberg equilibrium (HWE) tests in populations**

| Locus | Mt Elgon | | Baringo | Mau | Laikipia | Amudat | Moroto | Nakapiripirit |
| --- | --- | --- | --- | --- | --- | --- | --- | --- |
| KFOL2 | 0.128 | 0.996 | | 0.495 | 0.126 | **0.013** | **0.042** | 0.648 |
| KFOL13 | 0.996 | **0.014** | | **0.000** | **0.000** | 0.500 | **0.000** | **0.013** |
| KFOL17 | 0.092 | 0.009 | | 0.899 | 0.171 | **0.000** | 0.229 | 0.074 |
| KFOL24 | 0.315 | 0.185 | | 0.216 | **0.032** | **0.000** | **0.007** | 0.345 |
| KFOL28 | 0.068 | **0.001** | | **0.029** | 0.563 | **0.000** | **0.004** | **0.004** |
| KFOL30 | 0.958 | **0.002** | | 0.696 | 0.761 | **0.000** | 0.248 | **0.001** |
| KFOL37 | 0.543 | 0.273 | | 0.245 | 0.269 | 0.074 | **0.003** | 0.473 |
| KFOL42 | 1.000 | **0.000** | | 0.998 | **0.000** | **0.000** | **0.000** | **0.000** |
| KFOL47 | **0.000** | **0.000** | | **0.029** | **0.001** | **0.000** | **0.000** | 0.657 |
| KFOL48 | 0.946 | **0.011** | | **0.010** | 0.325 | **0.000** | **0.000** | **0.030** |

**Appendix** 4: Table 4 *p* values for genotypic linkage disequilibrium for each pair of loci across all populations

| Population | Locus#1 | Locus#2 | p value |
| --- | --- | --- | --- |
| Mt Elgon | KFOL2 | KFOL13 | 0.121 |
| Mt Elgon | KFOL2 | KFOL17 | 0.595 |
| Mt Elgon | KFOL13 | KFOL17 | 0.891 |
| Mt Elgon | KFOL2 | KFOL24 | 1.000 |
| Mt Elgon | KFOL13 | KFOL24 | 1.000 |
| Mt Elgon | KFOL17 | KFOL24 | 1.000 |
| Mt Elgon | KFOL2 | KFOL28 | 0.548 |
| Mt Elgon | KFOL13 | KFOL28 | 0.541 |
| Mt Elgon | KFOL17 | KFOL28 | 0.896 |
| Mt Elgon | KFOL24 | KFOL28 | 1.000 |
| Mt Elgon | KFOL2 | KFOL30 | 0.984 |
| Mt Elgon | KFOL13 | KFOL30 | 0.593 |
| Mt Elgon | KFOL17 | KFOL30 | 0.382 |
| Mt Elgon | KFOL24 | KFOL30 | 0.324 |
| Mt Elgon | KFOL28 | KFOL30 | 0.312 |
| Mt Elgon | KFOL2 | KFOL37 | 0.661 |
| Mt Elgon | KFOL13 | KFOL37 | 0.309 |
| Mt Elgon | KFOL17 | KFOL37 | 1.000 |
| Mt Elgon | KFOL24 | KFOL37 | 1.000 |
| Mt Elgon | KFOL28 | KFOL37 | 0.514 |
| Mt Elgon | KFOL30 | KFOL37 | 0.769 |
| Mt Elgon | KFOL2 | KFOL42 | 0.746 |
| Mt Elgon | KFOL13 | KFOL42 | 0.591 |
| Mt Elgon | KFOL17 | KFOL42 | 0.712 |
| Mt Elgon | KFOL24 | KFOL42 | 0.855 |
| Mt Elgon | KFOL28 | KFOL42 | 0.048 |
| Mt Elgon | KFOL30 | KFOL42 | 0.261 |
| Mt Elgon | KFOL37 | KFOL42 | 0.811 |
| Mt Elgon | KFOL2 | KFOL47 | 0.901 |
| Mt Elgon | KFOL13 | KFOL47 | 0.402 |
| Mt Elgon | KFOL17 | KFOL47 | 0.168 |
| Mt Elgon | KFOL24 | KFOL47 | 0.293 |
| Mt Elgon | KFOL28 | KFOL47 | 0.134 |
| Mt Elgon | KFOL30 | KFOL47 | 0.583 |
| Mt Elgon | KFOL37 | KFOL47 | 0.896 |
| Mt Elgon | KFOL42 | KFOL47 | 0.406 |
| Mt Elgon | KFOL2 | KFOL48 | 0.429 |
| Mt Elgon | KFOL13 | KFOL48 | 0.343 |
| Mt Elgon | KFOL17 | KFOL48 | 0.942 |
| Mt Elgon | KFOL24 | KFOL48 | 0.044 |
| Mt Elgon | KFOL28 | KFOL48 | 0.934 |
| Mt Elgon | KFOL30 | KFOL48 | 0.172 |
| Mt Elgon | KFOL37 | KFOL48 | 0.623 |
| Mt Elgon | KFOL42 | KFOL48 | 0.768 |
| Mt Elgon | KFOL47 | KFOL48 | 0.718 |
| Baringo | KFOL2 | KFOL13 | 0.380 |
| Baringo | KFOL2 | KFOL17 | 0.641 |
| Baringo | KFOL13 | KFOL17 | 0.553 |
| Baringo | KFOL2 | KFOL24 | 0.985 |
| Baringo | KFOL13 | KFOL24 | 0.843 |
| Baringo | KFOL17 | KFOL24 | 0.098 |
| Baringo | KFOL2 | KFOL28 | 0.092 |
| Baringo | KFOL13 | KFOL28 | 0.415 |
| Baringo | KFOL17 | KFOL28 | 0.116 |
| Baringo | KFOL24 | KFOL28 | 0.156 |
| Baringo | KFOL2 | KFOL30 | 0.392 |
| Baringo | KFOL13 | KFOL30 | 0.805 |
| Baringo | KFOL17 | KFOL30 | 0.331 |
| Baringo | KFOL24 | KFOL30 | 0.010 |
| Baringo | KFOL28 | KFOL30 | 0.054 |
| Baringo | KFOL2 | KFOL37 | 0.076 |
| Baringo | KFOL13 | KFOL37 | 0.850 |
| Baringo | KFOL17 | KFOL37 | 1.000 |
| Baringo | KFOL24 | KFOL37 | 0.483 |
| Baringo | KFOL28 | KFOL37 | 0.481 |
| Baringo | KFOL30 | KFOL37 | 0.756 |
| Baringo | KFOL2 | KFOL42 | 0.006 |
| Baringo | KFOL13 | KFOL42 | 0.602 |
| Baringo | KFOL17 | KFOL42 | 0.228 |
| Baringo | KFOL24 | KFOL42 | 0.005 |
| Baringo | KFOL28 | KFOL42 | 0.029 |
| Baringo | KFOL30 | KFOL42 | 0.063 |
| Baringo | KFOL37 | KFOL42 | 0.124 |
| Baringo | KFOL2 | KFOL47 | 0.573 |
| Baringo | KFOL13 | KFOL47 | 0.207 |
| Baringo | KFOL17 | KFOL47 | 0.363 |
| Baringo | KFOL24 | KFOL47 | 0.000 |
| Baringo | KFOL28 | KFOL47 | 0.485 |
| Baringo | KFOL30 | KFOL47 | 0.663 |
| Baringo | KFOL37 | KFOL47 | 1.000 |
| Baringo | KFOL42 | KFOL47 | 0.067 |
| Baringo | KFOL2 | KFOL48 | 0.076 |
| Baringo | KFOL13 | KFOL48 | 0.013 |
| Baringo | KFOL17 | KFOL48 | 0.456 |
| Baringo | KFOL24 | KFOL48 | 0.265 |
| Baringo | KFOL28 | KFOL48 | 0.006 |
| Baringo | KFOL30 | KFOL48 | 0.012 |
| Baringo | KFOL37 | KFOL48 | 0.293 |
| Baringo | KFOL42 | KFOL48 | 0.013 |
| Baringo | KFOL47 | KFOL48 | 0.926 |
| Mau | KFOL2 | KFOL13 | 0.385 |
| Mau | KFOL2 | KFOL17 | 0.639 |
| Mau | KFOL13 | KFOL17 | 0.263 |
| Mau | KFOL2 | KFOL24 | 0.909 |
| Mau | KFOL13 | KFOL24 | 0.792 |
| Mau | KFOL17 | KFOL24 | 1.000 |
| Mau | KFOL2 | KFOL28 | 0.531 |
| Mau | KFOL13 | KFOL28 | 0.021 |
| Mau | KFOL17 | KFOL28 | 1.000 |
| Mau | KFOL24 | KFOL28 | 0.175 |
| Mau | KFOL2 | KFOL30 | 0.276 |
| Mau | KFOL13 | KFOL30 | 0.186 |
| Mau | KFOL17 | KFOL30 | 0.000 |
| Mau | KFOL24 | KFOL30 | 0.518 |
| Mau | KFOL28 | KFOL30 | 0.904 |
| Mau | KFOL2 | KFOL37 | 0.061 |
| Mau | KFOL13 | KFOL37 | 0.846 |
| Mau | KFOL17 | KFOL37 | 0.060 |
| Mau | KFOL24 | KFOL37 | 0.423 |
| Mau | KFOL28 | KFOL37 | 0.489 |
| Mau | KFOL30 | KFOL37 | 0.293 |
| Mau | KFOL2 | KFOL42 | 0.576 |
| Mau | KFOL13 | KFOL42 | 1.000 |
| Mau | KFOL17 | KFOL42 | 0.889 |
| Mau | KFOL24 | KFOL42 | 0.574 |
| Mau | KFOL28 | KFOL42 | 0.883 |
| Mau | KFOL30 | KFOL42 | 1.000 |
| Mau | KFOL37 | KFOL42 | 0.554 |
| Mau | KFOL2 | KFOL47 | 0.016 |
| Mau | KFOL13 | KFOL47 | 0.143 |
| Mau | KFOL17 | KFOL47 | 1.000 |
| Mau | KFOL24 | KFOL47 | 0.719 |
| Mau | KFOL28 | KFOL47 | 0.017 |
| Mau | KFOL30 | KFOL47 | 0.609 |
| Mau | KFOL37 | KFOL47 | 0.616 |
| Mau | KFOL42 | KFOL47 | 0.680 |
| Mau | KFOL2 | KFOL48 | 0.211 |
| Mau | KFOL13 | KFOL48 | 0.008 |
| Mau | KFOL17 | KFOL48 | 0.445 |
| Mau | KFOL24 | KFOL48 | 0.612 |
| Mau | KFOL28 | KFOL48 | 0.358 |
| Mau | KFOL30 | KFOL48 | 0.161 |
| Mau | KFOL37 | KFOL48 | 0.189 |
| Mau | KFOL42 | KFOL48 | 1.000 |
| Mau | KFOL47 | KFOL48 | 0.004 |
| Laikipia | KFOL2 | KFOL13 | 0.039 |
| Laikipia | KFOL2 | KFOL17 | 0.237 |
| Laikipia | KFOL13 | KFOL17 | 0.108 |
| Laikipia | KFOL2 | KFOL24 | 0.683 |
| Laikipia | KFOL13 | KFOL24 | 0.269 |
| Laikipia | KFOL17 | KFOL24 | 0.878 |
| Laikipia | KFOL2 | KFOL28 | 0.162 |
| Laikipia | KFOL13 | KFOL28 | 0.433 |
| Laikipia | KFOL17 | KFOL28 | 0.715 |
| Laikipia | KFOL24 | KFOL28 | 0.030 |
| Laikipia | KFOL2 | KFOL30 | 0.466 |
| Laikipia | KFOL13 | KFOL30 | 0.447 |
| Laikipia | KFOL17 | KFOL30 | 0.015 |
| Laikipia | KFOL24 | KFOL30 | 0.646 |
| Laikipia | KFOL28 | KFOL30 | 0.360 |
| Laikipia | KFOL2 | KFOL37 | 0.244 |
| Laikipia | KFOL13 | KFOL37 | 0.076 |
| Laikipia | KFOL17 | KFOL37 | 0.607 |
| Laikipia | KFOL24 | KFOL37 | 0.963 |
| Laikipia | KFOL28 | KFOL37 | 0.269 |
| Laikipia | KFOL30 | KFOL37 | 0.489 |
| Laikipia | KFOL2 | KFOL42 | 0.480 |
| Laikipia | KFOL13 | KFOL42 | 0.278 |
| Laikipia | KFOL17 | KFOL42 | 0.318 |
| Laikipia | KFOL24 | KFOL42 | 0.690 |
| Laikipia | KFOL28 | KFOL42 | 0.185 |
| Laikipia | KFOL30 | KFOL42 | 0.778 |
| Laikipia | KFOL37 | KFOL42 | 0.963 |
| Laikipia | KFOL2 | KFOL47 | 0.386 |
| Laikipia | KFOL13 | KFOL47 | 0.107 |
| Laikipia | KFOL17 | KFOL47 | 0.321 |
| Laikipia | KFOL24 | KFOL47 | 0.306 |
| Laikipia | KFOL28 | KFOL47 | 0.919 |
| Laikipia | KFOL30 | KFOL47 | 0.219 |
| Laikipia | KFOL37 | KFOL47 | 0.930 |
| Laikipia | KFOL42 | KFOL47 | 0.452 |
| Laikipia | KFOL2 | KFOL48 | 0.415 |
| Laikipia | KFOL13 | KFOL48 | 0.523 |
| Laikipia | KFOL17 | KFOL48 | 0.759 |
| Laikipia | KFOL24 | KFOL48 | 0.645 |
| Laikipia | KFOL28 | KFOL48 | 0.985 |
| Laikipia | KFOL30 | KFOL48 | 0.094 |
| Laikipia | KFOL37 | KFOL48 | 0.026 |
| Laikipia | KFOL42 | KFOL48 | 1.000 |
| Laikipia | KFOL47 | KFOL48 | 0.018 |
| Amudat | KFOL2 | KFOL13 | 0.791 |
| Amudat | KFOL2 | KFOL17 | 0.000 |
| Amudat | KFOL13 | KFOL17 | 0.111 |
| Amudat | KFOL2 | KFOL24 | 0.626 |
| Amudat | KFOL13 | KFOL24 | 0.013 |
| Amudat | KFOL17 | KFOL24 | 0.008 |
| Amudat | KFOL2 | KFOL28 | 1.000 |
| Amudat | KFOL13 | KFOL28 | 1.000 |
| Amudat | KFOL17 | KFOL28 | 0.475 |
| Amudat | KFOL24 | KFOL28 | 0.109 |
| Amudat | KFOL2 | KFOL30 | 0.106 |
| Amudat | KFOL13 | KFOL30 | 0.363 |
| Amudat | KFOL17 | KFOL30 | 0.024 |
| Amudat | KFOL24 | KFOL30 | 0.000 |
| Amudat | KFOL28 | KFOL30 | 0.167 |
| Amudat | KFOL2 | KFOL37 | 0.254 |
| Amudat | KFOL13 | KFOL37 | 0.759 |
| Amudat | KFOL17 | KFOL37 | 0.017 |
| Amudat | KFOL24 | KFOL37 | 0.167 |
| Amudat | KFOL28 | KFOL37 | - |
| Amudat | KFOL30 | KFOL37 | 0.129 |
| Amudat | KFOL2 | KFOL42 | 0.621 |
| Amudat | KFOL13 | KFOL42 | 1.000 |
| Amudat | KFOL17 | KFOL42 | 0.008 |
| Amudat | KFOL24 | KFOL42 | 0.315 |
| Amudat | KFOL28 | KFOL42 | 0.083 |
| Amudat | KFOL30 | KFOL42 | 0.007 |
| Amudat | KFOL37 | KFOL42 | 0.371 |
| Amudat | KFOL2 | KFOL47 | 0.020 |
| Amudat | KFOL13 | KFOL47 | 0.430 |
| Amudat | KFOL17 | KFOL47 | 0.000 |
| Amudat | KFOL24 | KFOL47 | 0.000 |
| Amudat | KFOL28 | KFOL47 | 0.239 |
| Amudat | KFOL30 | KFOL47 | 0.000 |
| Amudat | KFOL37 | KFOL47 | 0.011 |
| Amudat | KFOL42 | KFOL47 | 0.081 |
| Amudat | KFOL2 | KFOL48 | 0.196 |
| Amudat | KFOL13 | KFOL48 | 0.109 |
| Amudat | KFOL17 | KFOL48 | 0.000 |
| Amudat | KFOL24 | KFOL48 | 0.004 |
| Amudat | KFOL28 | KFOL48 | 0.065 |
| Amudat | KFOL30 | KFOL48 | 0.000 |
| Amudat | KFOL37 | KFOL48 | 0.062 |
| Amudat | KFOL42 | KFOL48 | 0.054 |
| Amudat | KFOL47 | KFOL48 | 0.218 |
| Moroto | KFOL2 | KFOL13 | 0.231 |
| Moroto | KFOL2 | KFOL17 | 0.093 |
| Moroto | KFOL13 | KFOL17 | 0.031 |
| Moroto | KFOL2 | KFOL24 | 0.475 |
| Moroto | KFOL13 | KFOL24 | 0.192 |
| Moroto | KFOL17 | KFOL24 | 0.035 |
| Moroto | KFOL2 | KFOL28 | 0.184 |
| Moroto | KFOL13 | KFOL28 | 0.111 |
| Moroto | KFOL17 | KFOL28 | 0.197 |
| Moroto | KFOL24 | KFOL28 | 0.029 |
| Moroto | KFOL2 | KFOL30 | 1.000 |
| Moroto | KFOL13 | KFOL30 | 0.622 |
| Moroto | KFOL17 | KFOL30 | 0.011 |
| Moroto | KFOL24 | KFOL30 | 0.012 |
| Moroto | KFOL28 | KFOL30 | 0.089 |
| Moroto | KFOL2 | KFOL37 | 0.509 |
| Moroto | KFOL13 | KFOL37 | 0.214 |
| Moroto | KFOL17 | KFOL37 | 0.022 |
| Moroto | KFOL24 | KFOL37 | 0.002 |
| Moroto | KFOL28 | KFOL37 | 0.002 |
| Moroto | KFOL30 | KFOL37 | 0.000 |
| Moroto | KFOL2 | KFOL42 | 0.360 |
| Moroto | KFOL13 | KFOL42 | 0.320 |
| Moroto | KFOL17 | KFOL42 | 0.566 |
| Moroto | KFOL24 | KFOL42 | 0.014 |
| Moroto | KFOL28 | KFOL42 | 0.001 |
| Moroto | KFOL30 | KFOL42 | 0.002 |
| Moroto | KFOL37 | KFOL42 | 0.001 |
| Moroto | KFOL2 | KFOL47 | 0.712 |
| Moroto | KFOL13 | KFOL47 | 0.511 |
| Moroto | KFOL17 | KFOL47 | 0.239 |
| Moroto | KFOL24 | KFOL47 | 0.032 |
| Moroto | KFOL28 | KFOL47 | 0.000 |
| Moroto | KFOL30 | KFOL47 | 0.000 |
| Moroto | KFOL37 | KFOL47 | 0.113 |
| Moroto | KFOL42 | KFOL47 | 0.001 |
| Moroto | KFOL2 | KFOL48 | 0.752 |
| Moroto | KFOL13 | KFOL48 | 0.045 |
| Moroto | KFOL17 | KFOL48 | 0.717 |
| Moroto | KFOL24 | KFOL48 | 0.274 |
| Moroto | KFOL28 | KFOL48 | 0.029 |
| Moroto | KFOL30 | KFOL48 | 0.000 |
| Moroto | KFOL37 | KFOL48 | 0.059 |
| Moroto | KFOL42 | KFOL48 | 0.000 |
| Moroto | KFOL47 | KFOL48 | 0.000 |
| Nakapiripirit | KFOL2 | KFOL13 | 0.717 |
| Nakapiripirit | KFOL2 | KFOL17 | 0.570 |
| Nakapiripirit | KFOL13 | KFOL17 | 0.393 |
| Nakapiripirit | KFOL2 | KFOL24 | 0.429 |
| Nakapiripirit | KFOL13 | KFOL24 | 0.270 |
| Nakapiripirit | KFOL17 | KFOL24 | 0.257 |
| Nakapiripirit | KFOL2 | KFOL28 | - |
| Nakapiripirit | KFOL13 | KFOL28 | - |
| Nakapiripirit | KFOL17 | KFOL28 | - |
| Nakapiripirit | KFOL24 | KFOL28 | - |
| Nakapiripirit | KFOL2 | KFOL30 | 0.447 |
| Nakapiripirit | KFOL13 | KFOL30 | 0.553 |
| Nakapiripirit | KFOL17 | KFOL30 | 0.125 |
| Nakapiripirit | KFOL24 | KFOL30 | 0.486 |
| Nakapiripirit | KFOL28 | KFOL30 | - |
| Nakapiripirit | KFOL2 | KFOL37 | 0.520 |
| Nakapiripirit | KFOL13 | KFOL37 | 0.068 |
| Nakapiripirit | KFOL17 | KFOL37 | 0.789 |
| Nakapiripirit | KFOL24 | KFOL37 | 0.493 |
| Nakapiripirit | KFOL28 | KFOL37 | - |
| Nakapiripirit | KFOL30 | KFOL37 | 0.116 |
| Nakapiripirit | KFOL2 | KFOL42 | - |
| Nakapiripirit | KFOL13 | KFOL42 | - |
| Nakapiripirit | KFOL17 | KFOL42 | - |
| Nakapiripirit | KFOL24 | KFOL42 | - |
| Nakapiripirit | KFOL28 | KFOL42 | - |
| Nakapiripirit | KFOL30 | KFOL42 | - |
| Nakapiripirit | KFOL37 | KFOL42 | - |
| Nakapiripirit | KFOL2 | KFOL47 | 0.814 |
| Nakapiripirit | KFOL13 | KFOL47 | 0.254 |
| Nakapiripirit | KFOL17 | KFOL47 | 0.106 |
| Nakapiripirit | KFOL24 | KFOL47 | 0.009 |
| Nakapiripirit | KFOL28 | KFOL47 | - |
| Nakapiripirit | KFOL30 | KFOL47 | 0.337 |
| Nakapiripirit | KFOL37 | KFOL47 | 0.991 |
| Nakapiripirit | KFOL42 | KFOL47 | - |
| Nakapiripirit | KFOL2 | KFOL48 | 0.702 |
| Nakapiripirit | KFOL13 | KFOL48 | 0.141 |
| Nakapiripirit | KFOL17 | KFOL48 | 0.331 |
| Nakapiripirit | KFOL24 | KFOL48 | 0.000 |
| Nakapiripirit | KFOL28 | KFOL48 | - |
| Nakapiripirit | KFOL30 | KFOL48 | 0.200 |
| Nakapiripirit | KFOL37 | KFOL48 | 0.683 |
| Nakapiripirit | KFOL42 | KFOL48 | - |
| Nakapiripirit | KFOL47 | KFOL48 | 0.052 |

## Appendix 5 Table 5 Chi-square tests for Hardy-Weinberg equilibrium per loci per population

| **Pop** | **Locus** | **DF** | **Chi-Square** | **Probability** | **Significance** |
| --- | --- | --- | --- | --- | --- |
| Mt Elgon | KFOL2 | 6 | 9.930 | 0.128 | ns |
| Mt Elgon | KFOL13 | 15 | 4.457 | 0.996 | ns |
| Mt Elgon | KFOL17 | 91 | 109.382 | 0.092 | ns |
| Mt Elgon | KFOL24 | 91 | 96.966 | 0.315 | ns |
| Mt Elgon | KFOL28 | 15 | 23.824 | 0.068 | ns |
| Mt Elgon | KFOL30 | 36 | 22.782 | 0.958 | ns |
| Mt Elgon | KFOL37 | 105 | 102.765 | 0.543 | ns |
| Mt Elgon | KFOL42 | 10 | 0.860 | 1.000 | ns |
| Mt Elgon | KFOL47 | 28 | 70.581 | 0.000 | *** |
| Mt Elgon | KFOL48 | 10 | 4.024 | 0.946 | ns |
| Baringo | KFOL2 | 10 | 2.081 | 0.996 | ns |
| Baringo | KFOL13 | 36 | 57.235 | 0.014 | * |
| Baringo | KFOL17 | 78 | 110.628 | 0.009 | ** |
| Baringo | KFOL24 | 78 | 88.994 | 0.185 | ns |
| Baringo | KFOL28 | 28 | 56.292 | 0.001 | ** |
| Baringo | KFOL30 | 78 | 119.290 | 0.002 | ** |
| Baringo | KFOL37 | 120 | 128.879 | 0.273 | ns |
| Baringo | KFOL42 | 6 | 30.104 | 0.000 | *** |
| Baringo | KFOL47 | 66 | 221.895 | 0.000 | *** |
| Baringo | KFOL48 | 10 | 22.891 | 0.011 | * |
| Mau | KFOL2 | 10 | 9.392 | 0.495 | ns |
| Mau | KFOL13 | 36 | 74.537 | 0.000 | *** |
| Mau | KFOL17 | 91 | 74.271 | 0.899 | ns |
| Mau | KFOL24 | 45 | 52.151 | 0.216 | ns |
| Mau | KFOL28 | 10 | 20.078 | 0.029 | * |
| Mau | KFOL30 | 66 | 59.636 | 0.696 | ns |
| Mau | KFOL37 | 66 | 73.538 | 0.245 | ns |
| Mau | KFOL42 | 3 | 0.036 | 0.998 | ns |
| Mau | KFOL47 | 36 | 53.777 | 0.029 | * |
| Mau | KFOL48 | 3 | 11.302 | 0.010 | * |
| Laikipia | KFOL2 | 3 | 5.723 | 0.126 | ns |
| Laikipia | KFOL13 | 6 | 26.954 | 0.000 | *** |
| Laikipia | KFOL17 | 21 | 26.985 | 0.171 | ns |
| Laikipia | KFOL24 | 28 | 43.396 | 0.032 | * |
| Laikipia | KFOL28 | 10 | 8.682 | 0.563 | ns |
| Laikipia | KFOL30 | 36 | 29.717 | 0.761 | ns |
| Laikipia | KFOL37 | 10 | 12.238 | 0.269 | ns |
| Laikipia | KFOL42 | 3 | 26.059 | 0.000 | *** |
| Laikipia | KFOL47 | 10 | 31.414 | 0.001 | *** |
| Laikipia | KFOL48 | 1 | 0.968 | 0.325 | ns |
| Amudat | KFOL2 | 10 | 22.506 | 0.013 | * |
| Amudat | KFOL13 | 3 | 2.367 | 0.500 | ns |
| Amudat | KFOL17 | 190 | 275.102 | 0.000 | *** |
| Amudat | KFOL24 | 21 | 122.691 | 0.000 | *** |
| Amudat | KFOL28 | 3 | 120.000 | 0.000 | *** |
| Amudat | KFOL30 | 36 | 103.897 | 0.000 | *** |
| Amudat | KFOL37 | 10 | 17.028 | 0.074 | ns |
| Amudat | KFOL42 | 3 | 37.593 | 0.000 | *** |
| Amudat | KFOL47 | 91 | 182.581 | 0.000 | *** |
| Amudat | KFOL48 | 28 | 178.359 | 0.000 | *** |
| Moroto | KFOL2 | 3 | 8.210 | 0.042 | * |
| Moroto | KFOL13 | 3 | 40.000 | 0.000 | *** |
| Moroto | KFOL17 | 91 | 100.694 | 0.229 | ns |
| Moroto | KFOL24 | 78 | 112.041 | 0.007 | ** |
| Moroto | KFOL28 | 10 | 25.841 | 0.004 | ** |
| Moroto | KFOL30 | 21 | 24.980 | 0.248 | ns |
| Moroto | KFOL37 | 55 | 88.582 | 0.003 | ** |
| Moroto | KFOL42 | 6 | 33.645 | 0.000 | *** |
| Moroto | KFOL47 | 21 | 66.176 | 0.000 | *** |
| Moroto | KFOL48 | 10 | 40.274 | 0.000 | *** |
| Nakapiripirit | KFOL2 | 3 | 1.650 | 0.648 | ns |
| Nakapiripirit | KFOL13 | 15 | 29.728 | 0.013 | * |
| Nakapiripirit | KFOL17 | 120 | 143.111 | 0.074 | ns |
| Nakapiripirit | KFOL24 | 10 | 11.159 | 0.345 | ns |
| Nakapiripirit | KFOL28 | Monomorphic | |  |  |
| Nakapiripirit | KFOL30 | 28 | 57.371 | 0.001 | *** |
| Nakapiripirit | KFOL37 | 1 | 0.515 | 0.473 | ns |
| Nakapiripirit | KFOL42 | Monomorphic | |  |  |
| Nakapiripirit | KFOL47 | 36 | 32.049 | 0.657 | ns |
| Nakapiripirit | KFOL48 | 15 | 26.822 | 0.030 | * |

# Appendix 6 Table 6: Null alleles estimation among populations

| **Locus** | **Population** | **Estimate of null allele frequency** |
| --- | --- | --- |
| KFOL2 | Mt. Elgon | **0.11898** |
| KFOL13 | MtElgon | 0.00001 |
| KFOL17 | MtElgon | 0 |
| KFOL24 | MtElgon | 0.03888 |
| KFOL28 | MtElgon | 0 |
| KFOL30 | MtElgon | 0 |
| KFOL37 | MtElgon | 0 |
| KFOL42 | MtElgon | 0 |
| KFOL47 | MtElgon | **0.21987** |
| KFOL48 | MtElgon | 0 |
| KFOL2 | Baringo | 0.00001 |
| KFOL13 | Baringo | **0.16877** |
| KFOL17 | Baringo | 0.07849 |
| KFOL24 | Baringo | 0.0505 |
| KFOL28 | Baringo | 0.02732 |
| KFOL30 | Baringo | 0.08346 |
| KFOL37 | Baringo | 0.01001 |
| KFOL42 | Baringo | **0.17229** |
| KFOL47 | Baringo | **0.29809** |
| KFOL48 | Baringo | 0.02315 |
| KFOL2 | Mau | 0 |
| KFOL13 | Mau | 0.05501 |
| KFOL17 | Mau | 0.00001 |
| KFOL24 | Mau | 0.01217 |
| KFOL28 | Mau | 0 |
| KFOL30 | Mau | 0.01044 |
| KFOL37 | Mau | 0.01678 |
| KFOL42 | Mau | 0.00003 |
| KFOL47 | Mau | 0.1394 |
| KFOL48 | Mau | 0.1264 |
| KFOL2 | Laikipia | **0.10528** |
| KFOL13 | Laikipia | 0.09696 |
| KFOL17 | Laikipia | 0.03128 |
| KFOL24 | Laikipia | 0 |
| KFOL28 | Laikipia | 0.09348 |
| KFOL30 | Laikipia | 0 |
| KFOL37 | Laikipia | 0.00001 |
| KFOL42 | Laikipia | **0.21716** |
| KFOL47 | Laikipia | **0.17752** |
| KFOL48 | Laikipia | 0.06013 |
| KFOL2 | Amudat | 0.05655 |
| KFOL13 | Amudat | 0.0445 |
| KFOL17 | Amudat | 0.09284 |
| KFOL24 | Amudat | 0.04367 |
| KFOL28 | Amudat | 0.00099 |
| KFOL30 | Amudat | **0.11288** |
| KFOL37 | Amudat | **0.1019** |
| KFOL42 | Amudat | **0.10718** |
| KFOL47 | Amudat | 0.00045 |
| KFOL48 | Amudat | **0.16624** |
| KFOL2 | Moroto | **0.11337** |
| KFOL13 | Moroto | **0.30436** |
| KFOL17 | Moroto | 0 |
| KFOL24 | Moroto | 0.06502 |
| KFOL28 | Moroto | 0.08748 |
| KFOL30 | Moroto | **0.17093** |
| KFOL37 | Moroto | 0.07272 |
| KFOL42 | Moroto | **0.23888** |
| KFOL47 | Moroto | **0.22315** |
| KFOL48 | Moroto | **0.27013** |
| KFOL2 | Nakapiripirit | 0 |
| KFOL13 | Nakapiripirit | **0.2069** |
| KFOL17 | Nakapiripirit | 0.03293 |
| KFOL24 | Nakapiripirit | 0.01878 |
| KFOL28 | Nakapiripirit | 0.001 |
| KFOL30 | Nakapiripirit | 0.05929 |
| KFOL37 | Nakapiripirit | 0.05525 |
| KFOL42 | Nakapiripirit | 0.001 |
| KFOL47 | Nakapiripirit | 0.01681 |
| KFOL48 | Nakapiripirit | 0 |

# Appendix 7: Table 7: Genetic diversity indices for the 10 loci over the seven studied populations

| Loci | Na | Ne | I | Ho | He | F | FST | Nm |
| --- | --- | --- | --- | --- | --- | --- | --- | --- |
| KFOL2 | 4.000 | 2.104 | 0.895 | 0.453 | 0.508 | 0.125 | 0.166 | 1.252 |
| KFOL13 | 5.714 | 2.316 | 1.020 | 0.340 | 0.526 | 0.345 | 0.317 | 0.538 |
| KFOL17 | 14.000 | 8.954 | 2.340 | 0.814 | 0.897 | 0.075 | 0.056 | 4.215 |
| KFOL24 | 10.000 | 5.854 | 1.833 | 0.711 | 0.780 | 0.072 | 0.155 | 1.359 |
| KFOL28 | 4.714 | 2.759 | 1.012 | 0.528 | 0.518 | 0.048 | 0.339 | 0.488 |
| KFOL30 | 9.571 | 4.057 | 1.642 | 0.620 | 0.737 | 0.145 | 0.124 | 1.774 |
| KFOL37 | 9.429 | 5.064 | 1.621 | 0.626 | 0.678 | 0.083 | 0.225 | 0.860 |
| KFOL42 | 3.286 | 1.455 | 0.478 | 0.123 | 0.245 | 0.398 | 0.570 | 0.188 |
| KFOL47 | 9.143 | 5.001 | 1.777 | 0.523 | 0.792 | 0.334 | 0.099 | 2.272 |
| KFOL48 | 4.857 | 2.284 | 0.970 | 0.368 | 0.503 | 0.254 | 0.252 | 0.741 |
| Mean | 7.471 | 3.985 | 1.359 | 0.511 | 0.618 | 0.187 | 0.230 | 1.369 |

# Appendix 8: Table 8 F statistics results

| Source | F-statistics | P(rand>=data) |
| --- | --- | --- |
| Among regions (UG/KE)  Among populations  Among individuals  Within individuals  Total | Frt = 0.168  Fsr = 0.118  Fst = 0.266  Fis = 0.196  Fit = 0.410  Nm = 0.690 | 0.000  0.000  0.000  0.000  0.001 |

**Appendix 9 Table 9: Pairwise FST and Nm values across populations in Uganda and Kenya**

| Pop1 | Pop 2 | FST | Nm | Pop size1 | Pop2 |
| --- | --- | --- | --- | --- | --- |
| Mt. Elgon | Baringo | 0.023 | 10.642 | 16 | 16 |
| Mt. Elgon | Mau | 0.035 | 6.859 | 16 | 16 |
| Baringo | Mau | 0.045 | 5.263 | 16 | 16 |
| Mt. Elgon | Laikipia | 0.067 | 3.476 | 16 | 16 |
| Baringo | Laikipia | 0.076 | 3.046 | 16 | 16 |
| Mau | Laikipia | 0.077 | 2.994 | 16 | 16 |
| Mt. Elgon | Amudat | 0.248 | 0.757 | 16 | 16 |
| Baringo | Amudat | 0.223 | 0.872 | 16 | 16 |
| Mau | Amudat | 0.264 | 0.699 | 16 | 16 |
| Laikipia | Amudat | 0.253 | 0.736 | 16 | 16 |
| Mt. Elgon | Moroto | 0.081 | 2.836 | 16 | 16 |
| Baringo | Moroto | 0.068 | 3.423 | 16 | 16 |
| Mau | Moroto | 0.095 | 2.370 | 16 | 16 |
| Laikipia | Moroto | 0.119 | 1.846 | 16 | 16 |
| Amudat | Moroto | 0.172 | 1.207 | 16 | 16 |
| Mt. Elgon | Nakapiripirit | 0.234 | 0.820 | 16 | 16 |
| Baringo | Nakapiripirit | 0.211 | 0.934 | 16 | 16 |
| Mau | Nakapiripirit | 0.246 | 0.767 | 16 | 16 |
| Laikipia | Nakapiripirit | 0.260 | 0.710 | 16 | 16 |
| Amudat | Nakapiripirit | 0.068 | 3.426 | 16 | 16 |
| Moroto | Nakapiripirit | 0.153 | 1.386 | 16 | 16 |

**Appendix 10: Table 10 Delta K values for the seven K populations proposed**

| **K** | **Reps** | **Mean LnP(K)** | **Stdev LnP(K)** | **Ln'(K)** | **|Ln''(K)|** | **Delta K** |
| --- | --- | --- | --- | --- | --- | --- |
| 1 | 10 | -8128.47 | 1.0078 | — | — | — |
| **2** | **10** | **-6500.31** | **0.9073** | **1628.16** | **1430.74** | **1576.893** |
| 3 | 10 | -6302.89 | 30.2817 | 197.42 | 27.6 | 0.911442 |
| 4 | 10 | -6133.07 | 30.2322 | 169.82 | 77.47 | 2.562498 |
| 5 | 10 | -6040.72 | 52.7917 | 92.35 | 244.5 | 4.631413 |
| 6 | 10 | -6192.87 | 870.1394 | -152.15 | 456.03 | 0.524088 |
| 7 | 10 | -6801.05 | 1499.783 | -608.18 | 1606.45 | 1.071122 |
| 8 | 10 | -5802.78 | 19.0209 | 998.27 | 1162.54 | 61.11905 |
| 9 | 10 | -5967.05 | 570.0538 | -164.27 | 408.53 | 0.716652 |
| 10 | 10 | -5722.79 | 167.4024 | 244.26 | — | — |


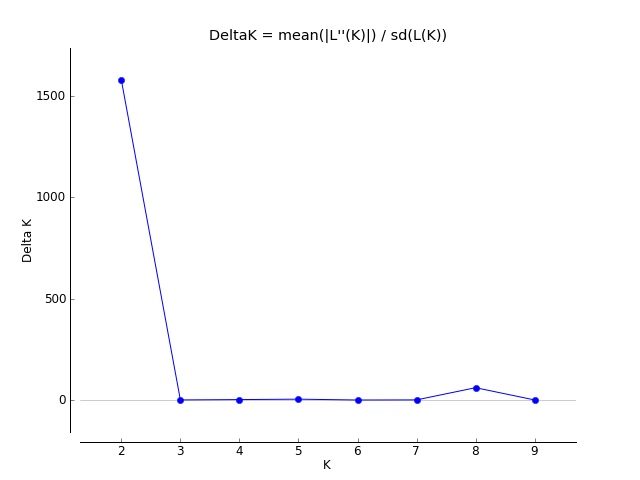

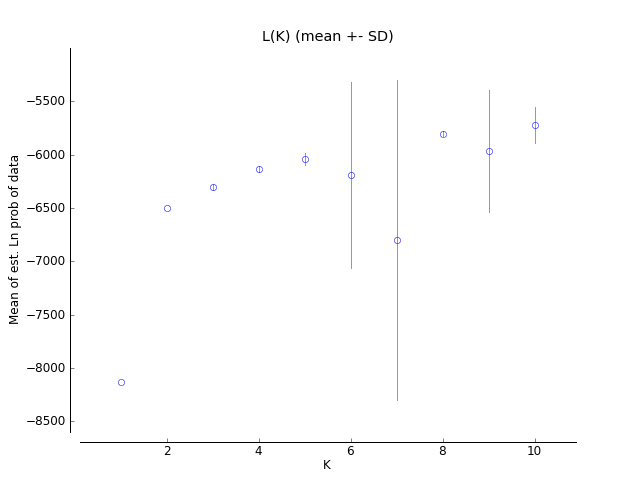


**Appendix 11 Criteria for detecting the optimum K values by Delta K values**

**Figure** 11*: Values of ΔK, with its modal value detecting a true K of the two groups (K = 2), (****b****) Log likelihood of the data (n = 210), L (K), as a function of K (the number of groups used to stratify the sample). For each K value, 10 independent runs were considered, and the data were averaged over the replicates.*


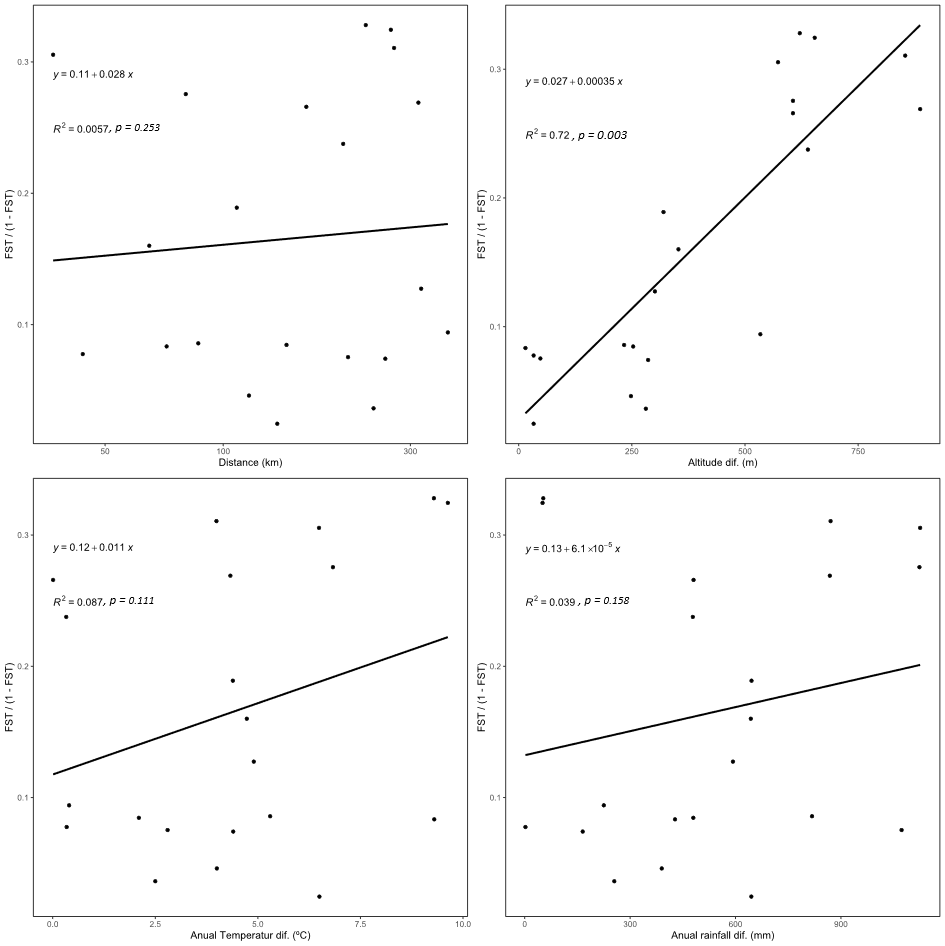
**Appendix 12: Results of Mantel Tests for isolation by distance, temperature, rainfall and altitude**

Figure 12. Isolation by distance, by altitude, temperature, and rainfall analyses. Mantel test p-values are provided for each comparison.

**Appendix 13 Barriers identified among sampled populations**


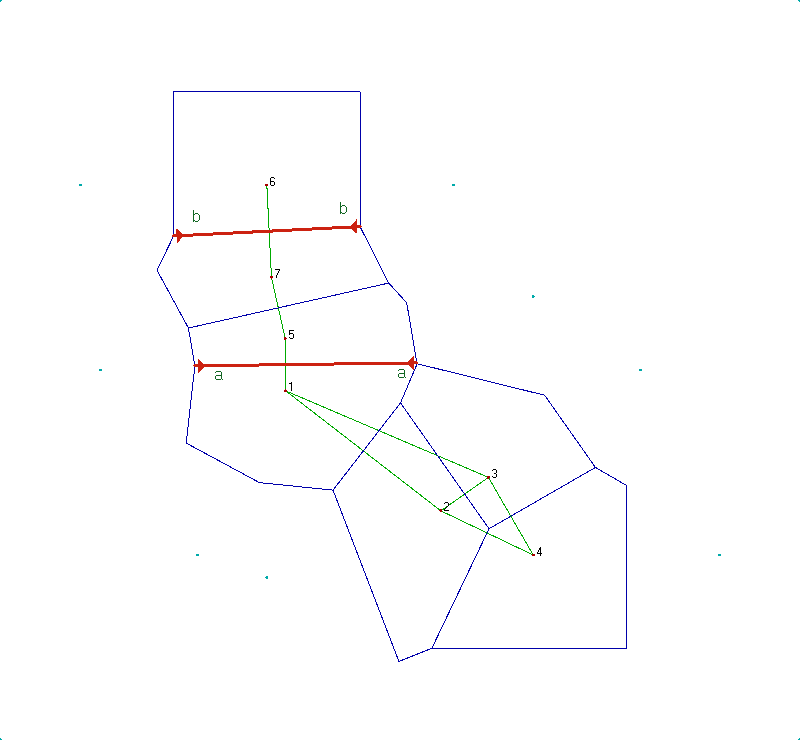

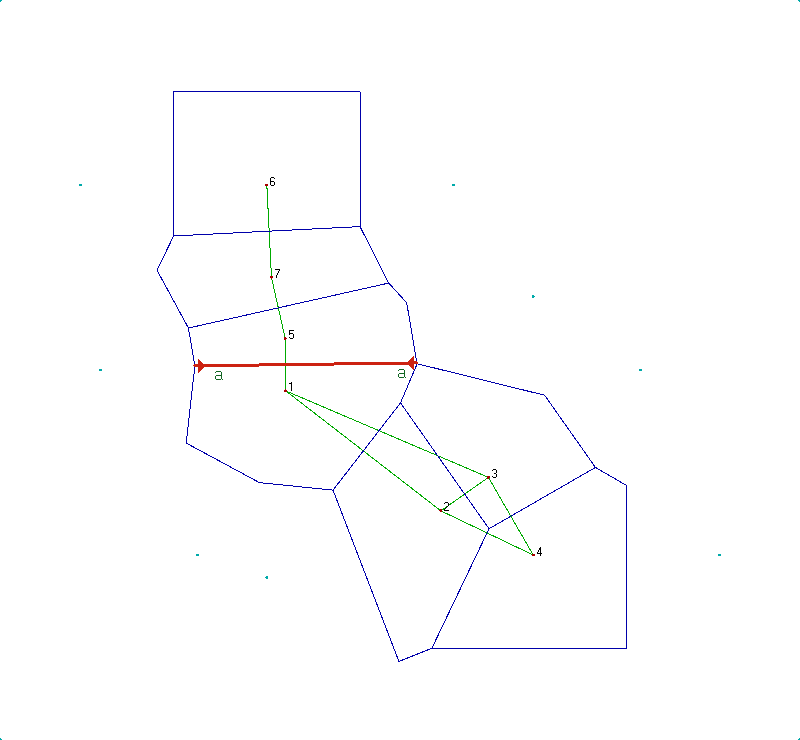

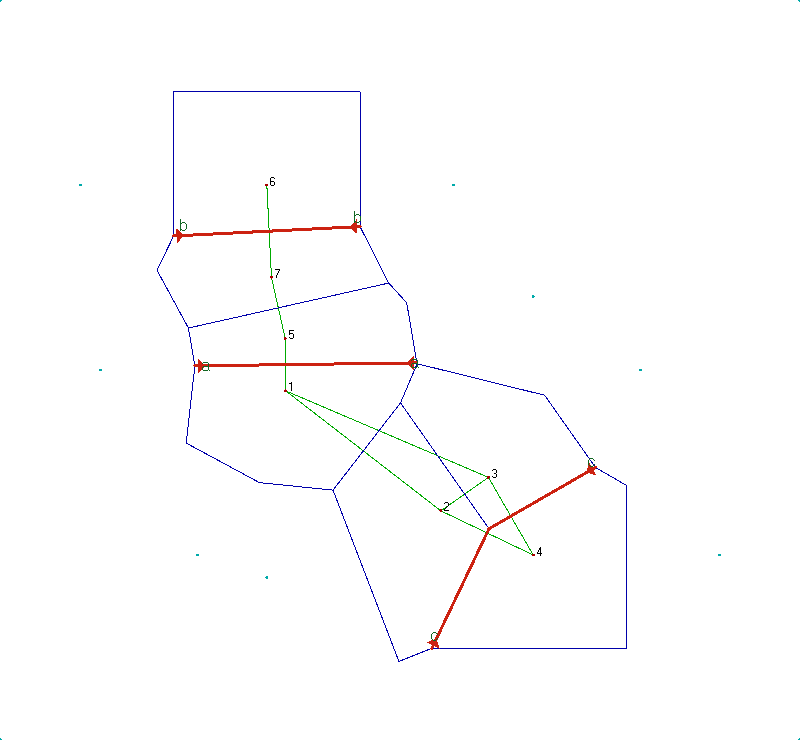

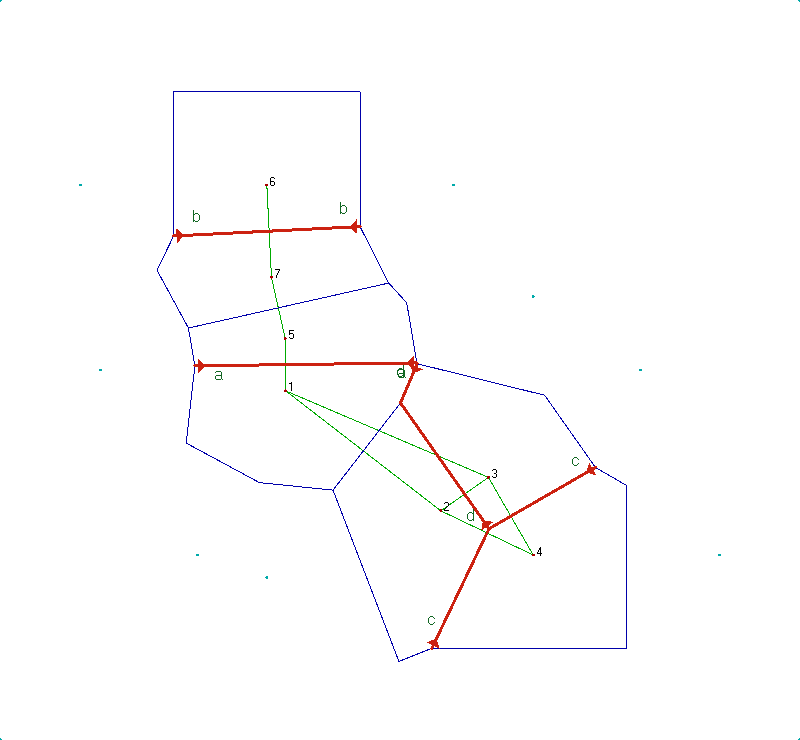


Figure 13. Maps showing the main barriers between the populations calculated with BARRIERS. Between 1 to four barriers were considered and named a, b, c, and d. Numbers correspond to the following populations: 1- Mt. Elgon; 2- Baringo; 3 – Mau; 4 – Laikipia; 5 – Amudat; 6 – Moroto; 7 – Nakapiripirit.

**Appendix 14: Migration rates across sink and source populations in Uganda and Kenya**

| **Sink** | **Source** | **Contemporary migration rate** | | **95% CI** |
| --- | --- | --- | --- | --- |
| Mt. Elgon | Mt. Elgon | 0.9218 | 0.0251 | |
| Baringo | Mt. Elgon | **0.2509** | **0.0235** | |
| Mau | Mt. Elgon | **0.2693** | **0.0217** | |
| Laikipia | Mt. Elgon | 0.0207 | 0.0161 | |
| Amudat | Mt. Elgon | 0.005 | 0.0049 | |
| Moroto | Mt. Elgon | 0.0125 | 0.012 | |
| Nakapiripirit | Mt. Elgon | 0.0144 | 0.0137 | |
| Mt. Elgon | Baringo | 0.0096 | 0.0092 | |
| Baringo | Baringo | **0.6762** | **0.0093** | |
| Mau | Baringo | 0.0096 | 0.0092 | |
| Laikipia | Baringo | 0.0102 | 0.0099 | |
| Amudat | Baringo | 0.005 | 0.0049 | |
| Moroto | Baringo | 0.0123 | 0.0119 | |
| Nakapiripirit | Baringo | 0.0143 | 0.0137 | |
| Mt. Elgon | Mau | 0.0096 | 0.0094 | |
| Baringo | Mau | 0.0091 | 0.0088 | |
| Mau | Mau | **0.676** | **0.0091** | |
| Laikipia | Mau | 0.0101 | 0.0098 | |
| Amudat | Mau | 0.0049 | 0.0049 | |
| Moroto | Mau | 0.0123 | 0.0118 | |
| Nakapiripirit | Mau | 0.0145 | 0.0138 | |
| Mt. Elgon | Laikipia | *0.0303* | *0.0181* | |
| Baringo | Laikipia | 0.0104 | 0.01 | |
| Mau | Laikipia | 0.0174 | 0.0128 | |
| Laikipia | Laikipia | **0.9287** | **0.0249** | |
| Amudat | Laikipia | 0.0099 | 0.0069 | |
| Moroto | Laikipia | **0.1856** | **0.0315** | |
| Nakapiripirit | Laikipia | 0.0145 | 0.0138 | |
| Mt. Elgon | Amudat | 0.0095 | 0.0092 | |
| Baringo | Amudat | 0.0093 | 0.0091 | |
| Mau | Amudat | 0.0091 | 0.0088 | |
| Laikipia | Amudat | 0.0101 | 0.0098 | |
| Amudat | Amudat | **0.9601** | **0.0131** | |
| Moroto | Amudat | 0.0132 | 0.0126 | |
| Nakapiripirit | Amudat | **0.1027** | **0.0316** | |
| Mt. Elgon | Moroto | 0.0096 | 0.0093 | |
| Baringo | Moroto | 0.0092 | 0.0089 | |
| Mau | Moroto | 0.0096 | 0.0093 | |
| Laikipia | Moroto | 0.0101 | 0.0098 | |
| Amudat | Moroto | 0.0049 | 0.0049 | |
| Moroto | Moroto | **0.679** | **0.0118** | |
| Nakapiripirit | Moroto | 0.0146 | 0.0139 | |
| Mt. Elgon | Nakapiripirit | 0.0095 | 0.0092 | |
| Baringo | Nakapiripirit | *0.0349* | *0.017* | |
| Mau | Nakapiripirit | 0.009 | 0.0087 | |
| Laikipia | Nakapiripirit | 0.0102 | 0.0098 | |
| Amudat | Nakapiripirit | 0.0101 | 0.007 | |
| Moroto | Nakapiripirit | *0.0851* | *0.028* | |
| Nakapiripirit | Nakapiripirit | **0.825** | **0.0342** | |
